# Effects of Copper or Zinc Organometallics on Cytotoxicity, DNA Damage and Epigenetic Changes in the HC-04 Human Liver Cell Line

**DOI:** 10.3390/ijms242115580

**Published:** 2023-10-25

**Authors:** Daniel Desaulniers, Gu Zhou, Andrew Stalker, Cathy Cummings-Lorbetskie

**Affiliations:** 1 Health Canada, Environmental Health Science and Research Bureau, Ottawa, ON K1A 0K9, Canada; daniel.desaulniers@hc-sc.gc.ca (D.D.);; 2 Health Canada, Regulatory Research Division, Biologics and Radiopharmaceutical Drugs Directorate, Ottawa, ON K1A 0K9, Canada

**Keywords:** organometallics, dithiocarbamates, copper, zinc, DNA methylation, histones, epigenetics, hepatocytes

## Abstract

Copper and zinc organometallics have multiple applications and many are considered “data-poor” because the available toxicological information is insufficient for comprehensive health risk assessments. To gain insight into the chemical prioritization and potential structure activity relationship, the current work compares the in vitro toxicity of nine “data-poor” chemicals to five structurally related chemicals and to positive DNA damage inducers (4-nitroquinoline-oxide, aflatoxin-B1). The HC-04 non-cancer human liver cell line was used to investigate the concentration–response effects (24 h and 72 h exposure) on cell proliferation, DNA damage (γH2AX and DNA unwinding assays), and epigenetic effects (global genome changes in DNA methylation and histone modifications using flow cytometry). The 24 h exposure screening data (DNA abundance and damage) suggest a toxicity hierarchy, starting with copper dimethyldithiocarbamate (CDMDC, CAS#137-29-1) > zinc diethyldithiocarbamate (ZDEDC, CAS#14324-55-1) > benzenediazonium, 4-chloro-2-nitro-, and tetrachlorozincate(2-) (2:1) (BDCN4CZ, CAS#14263-89-9); the other chemicals were less toxic and had alternate ranking positions depending on assays. The potency of CDMDC for inducing DNA damage was close to that of the human hepatocarcinogen aflatoxin-B1. Further investigation using sodium-DMDC (SDMDC, CAS#128-04-1), CDMDC and copper demonstrated the role of the interactions between copper and the DMDC organic moiety in generating a high level of CDMDC toxicity. In contrast, additive interactions were not observed with respect to the DNA methylation flow cytometry data in 72 h exposure experiments. They revealed chemical-specific effects, with hypo and hypermethylation induced by copper chloride (CuCl_2_, CAS#10125-13-0) and zinc-DMDC (ZDMDC, CAS#137-30-4), respectively, but did not show any significant effect of CDMDC or SDMDC. Histone-3 hypoacetylation was a sensitive flow cytometry marker of 24 h exposure to CDMDC. This study can provide insights regarding the prioritization of chemicals for future study, with the aim being to mitigate chemical hazards.

## 1. Introduction

Copper and zinc organometallics (CuOM, ZnOM) have multiple applications, and many are considered “data-poor” because the available toxicological information is insufficient to achieve detailed health risk assessments. Organometallics include a metal ion either to provide redox potential (e.g., Cu^2+^, Fe^2+^) or molecular structural conformation (e.g., Zn^2+^, which does not have redox potential). The organic moiety provides additional chemical properties and molecular interaction potential. Simple molecules containing copper or zinc are used as pesticides, fungicides, and as agents in chemical reactions (e.g., copper sulphate, CuCl_2_* (the symbol * identifies “data-poor” chemicals in the introduction and in Table 1), copper bromide (CuBr_2_*, zinc chloride (ZnCl_2_), while more complex molecules are used as dietary supplements [copper, bis(D-gluconato-O1,O2)-] (CuDg*), in personal care products and cleaning agents (10-undecenoic acid, zinc salt*; zinc phenolsulfonate (ZPS*); zinc p-toluenesulfonate hydrate (ZpTS*); benzoic acid, 4-(1,1-dimethylethyl)-, zinc salt (2:1) (BADZ*), in wood preservatives (copper, C6-19-branched carboxylate naphthenate complexes), in azo dyes [benzenediazonium, 3-methyl-4-(1-pyrrolidinyl)-, trichlorozincate(1-)] (BDMP3CZ*), and in paint and pigments [copper, [1-[[(2-hydroxyphenyl)imino]methyl]-2-naphthalenolato(2-)-n]*. Dithiocarbamate (DTC) family members can include Cu^2+^ or Zn^2+^, but also Mn^2+^ or Fe^2+^; these generate organometallic biocides such as Ziram, Maneb and Ferbam [1,2]. The United States has been reported to annually use nearly 1400 metric tons of SDMDTC in fungicides to treat plants, in insecticides, rodent repellents, slimicides [3], and as a replacement for the chromated copper-arsenate wood preservative [3,4]. There is currently interest in DTC analogues for multiple medicinal applications [1,2,5,6]. Here, the toxic effects of CDMDTC* are compared to those induced by ZDMDC (commercial name Ziram), SDMDC, and ZDEDC*.

Zinc organometallics include some azo dyes such as the benzenediazonium-containing molecules BDMP3CZ* and BDCN4CZ*. The benzenediazonium (BD) salts are known to be carcinogenic in mice [7]. The BD ion binds to DNA and RNA, generating covalent adducts in vitro and in vivo, among which 8-(phenylazo)guanine is a major DNA adduct [8]. The metabolism of dyes such as Sudan-I, leads to the formation of BD, which is genotoxic in HepG2 cells [9,10] and carcinogenic [8]. Therefore, the effects of BDMP3CZ* and BDCN4CZ* are compared here to the dye Sudan-I.

Zinc and copper ions, however, are essential to normal physiological functions. For example, the metalloenzyme copper/zinc-superoxide dismutase (Cu/Zn-SOD) confers cellular protection against reactive oxygen species that otherwise can induce lipid peroxidation, protein and DNA damage. Cu^2+^ is a cofactor for other enzymes such as cytochrome c oxidase, dopamine β-hydroxylase, tyrosinase and ceruloplasmin. Zn^2+^ is responsible for the conformation of the largest family of transcription factors interacting with DNA for the regulation of gene transcription. The human genome encodes approximately 3000 zinc proteins [11]. Therefore, there is generally more concern about dietary deficiencies rather than excess exposure to these essential elements, although copper deficiency and excess can both produce toxicity and adverse health effects [12,13,14]. Occupational exposure to copper has been reported to be genotoxic, inducing chromosome aberrations, an increased frequency of micronuclei in peripheral blood leukocytes, and DNA damage, detected via comet assays [15,16]. The zinc-buffering capacity of a cell and cellular environment determines whether zinc is cytoprotective or cytotoxic [17]. Metallothioneins provide zinc-buffering capacity and exist as a family of redox proteins [at least 12 [11]] that, in vivo, can bind mainly Zn^2+^, Cu+, Cd^2+^ and Hg^2+^; however, they are found mostly containing zinc under physiological conditions [18]. The adverse effects of Zn^2+^ can occur due to its avidity to protein, and free zinc in excess can cause protein misfolding [17,19].

Human carcinogens possess 10 key characteristics, which include being genotoxic, having the ability to alter DNA repair or to cause genomic instability, and being able to induce epigenetic alterations [20,21]. Irrespective of the mechanisms that initiate cancer development (genotoxic or non-genotoxic events, direct or indirect carcinogens), a common event in cancers is the induction of epigenetic changes [22]. In human liver cells in vitro, copper was reported to bind histone molecules and to induce epigenetic imbalances such as reducing histone acetyltransferase (HAT) activity [23] without affecting histone deacetylases (HDAC) [24]. The inhibition of HAT activities was observed in Hep3B cells treated with Cu^2+^-phenanthroline [25] and in HL-60 cells exposed to Cu^2+^-pyrrolidine dithiocarbamate [26]. The copper-induced inhibition of HAT activities was partly reverted by the addition of reactive oxygen species (ROS) scavengers, supporting the adverse effects of ROS on HAT activities [27]. In tx-j mouse, hepatic copper accumulation was associated with inflammation, the down-regulation of DNA methyltransferase-3b and global DNA hypomethylation [28].

Consequently, the current work aims to compare CuOM and ZnOM exposures to control chemicals, assessing their ability to induce DNA methylation changes and histone modifications, their toxicity based on the measurement of DNA abundance, their induction of DNA damage using γH2AX assays to detect double-strand breaks (DSB) [29], and DNA alkaline unwinding assays sensitive to both DNA single-strand breaks (SSB) and DSB [30]. This work also aims to investigate how DNA damage is affected by the interaction between metal ions and their organometallic moiety by mixing a less toxic DMDC compound (SDMDC) with two sources of copper(II) ion: CuCl_2_ and CuDg.

## 2. Results

### 2.1. Cytotoxicity and DNA Damage Induction

**Figure 1:**
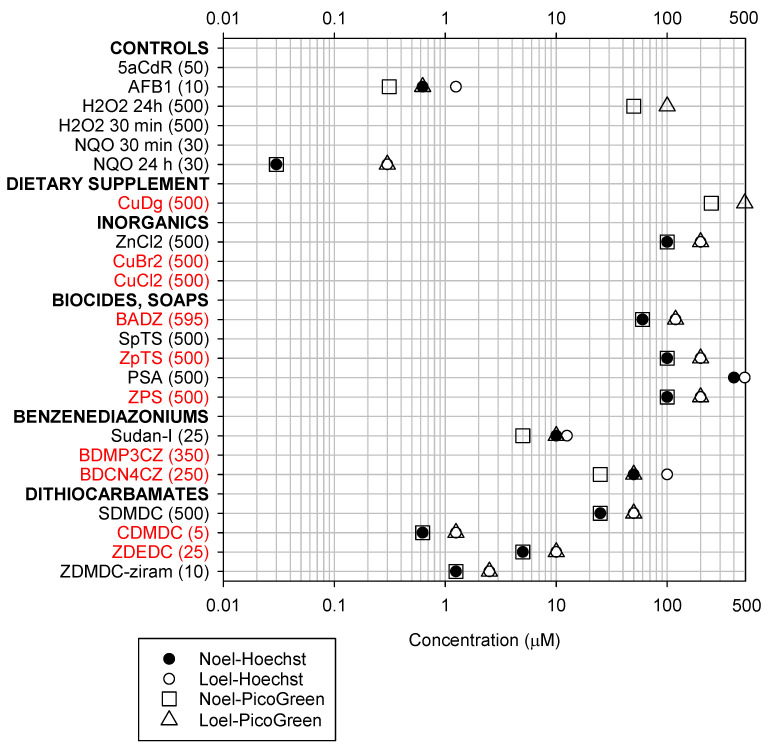
The effect of treatment with different copper and zinc chemicals on the reduction in cellular abundance. The no-observable-effect levels (NOELs: black circle, and square) and lowest-observable-effect levels (LOELs: open circle and triangle) were derived from concentration–response experiments. As a proxy for cellular abundance, DNA abundance data were obtained from Hoechst or PicoGreen DNA staining from the γH2AX or SSF assays, respectively. Some data were from only the Hoechst (phenolsulfonic acid (PSA)) or the PicoGreen assays (hydrogen peroxide (H_2_O_2_) 24 h, copper(II) D-gluconate (CuDg)), while other listed chemicals did not show any symbols because they did not induce significant changes over the tested concentration range. The largest concentration tested is provided within the parenthesis beside the chemical name. No NOEL symbols are provided when the LOEL was the smallest concentration tested. Chemicals in red font are “data-poor” chemicals.

**Figure 2:**
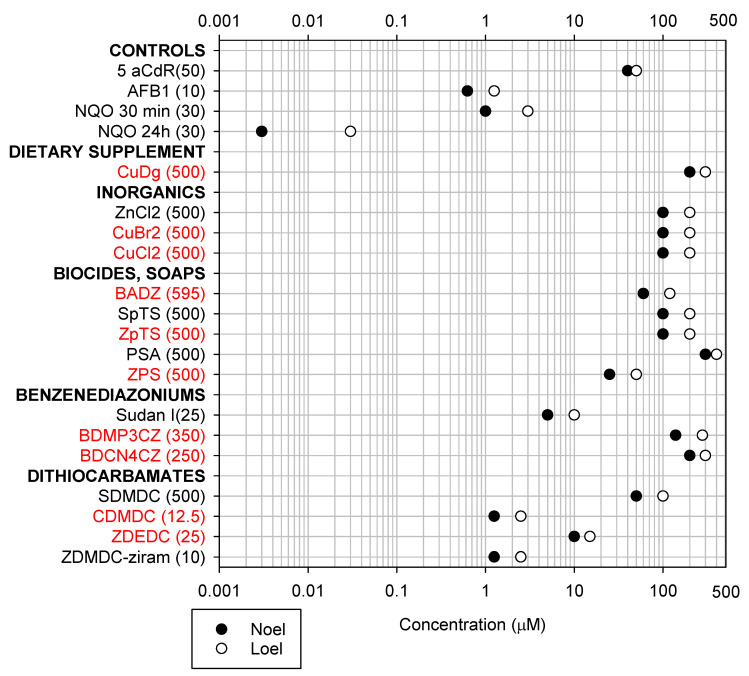
The effect of treatment with different copper and zinc chemicals on the elevation of pH2AX relative to DNA abundance (pH2AX/DNA). Concentration–response experiments generated no-observable-effect levels (NOELs, black circle) and lowest-observable-effect levels (LOELs, open circle). The largest concentration tested is provided within the parenthesis beside the chemical name. Chemicals in red font are “data-poor” chemicals.

**Figure 3:**
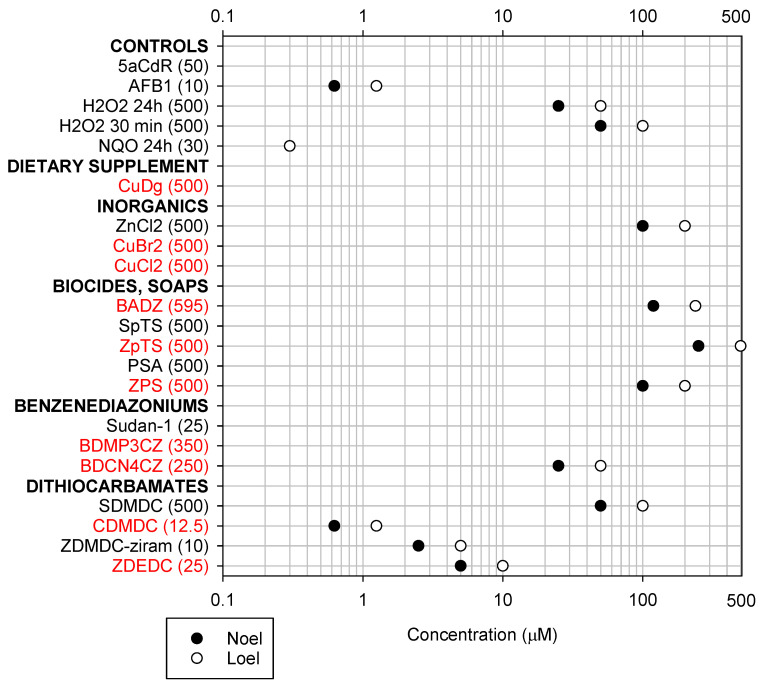
The effect of treatment with different copper and zinc chemicals on the elevation of strand scission factor (SSF) values as an indication of DNA damage. No-observable-effect levels (NOELs, black circle) and lowest-observable-effect levels (LOELs, open circle) were derived from concentration–response experiments. The largest concentration tested is provided within the parenthesis beside the chemical name. NOEL was not provided for 4-nitroquinoline-oxide (NQO), as the smallest concentration tested was the LOEL. Some chemicals do not have any symbols because they did not induce a significant change at the highest concentration. Chemicals in red font are “data-poor” chemicals.

Figure 1, Figure 2 and Figure 3 present the lowest-observable-effect levels (LOELs; the smallest concentration inducing a statistically significant effect) and no-observable-effect levels (NOELs; the concentration that precedes the LOEL) affecting the abundance of DNA, phosphorylated (p)H2AX/DNA, and the DNA damage strand scission factor (SSF), respectively. As shown in Figure 1, Aflatoxin B1 (AFB1) induced decreases in DNA abundance at similar NOELs and LOELs of 0.625 µM and 1.25 µM using the Hoechst dye, and 0.3125 µM and 0.625 µM using the PicoGreen dye, respectively. AFB1 induced DNA damage at NOELs and LOELs of 0.625 µM and 1.25 µM, respectively, based on both the pH2AX/DNA (Figure 2) and SSF assays (Figure 3). These figures (1 to 3) summarize large datasets for screening chemical toxicity. All chemicals were tested in a minimum of three different experiments. Supplementary Figure S2 shows how the NOELs and LOELs were obtained using AFB1 as an example. Some chemicals induced proliferative effects at low concentrations before inducing toxicity at higher concentrations; these NOELs and LOELs are presented in Supplementary Figure S9.

Among all zinc organometallics, ZDMDC was the most toxic, reducing DNA abundance and increasing the pH2AX/DNA ratio at 2.5 µM (Figure 1 and Figure 2). Among the inorganics (ZnCl_2_, CuCl_2_, CuBr_2_), only ZnCl_2_ decreased the DNA abundance (Figure 1) and increased the SSF (Figure 3). Zinc-based biocides exerted effects generally at similar concentrations (Figure 1, Figure 2 and Figure 3), with ZPS inducing the lowest pH2AX/total H2AX LOEL (50 µM) in that group (Figure 2). Among the benzenediazonium dyes, Sudan-I was the most toxic, inducing a decrease in DNA abundance at 10 µM (Figure 1).

Among all organometallics, CDMDC was the most toxic. CDMDC generated the lowest NOEL and LOEL values for decrease in DNA abundance (0.625 µM and 1.25 µM, Figure 1) and DNA damage; as measured by the pH2AX/DNA (1.25 µM and 2.5 µM, Figure 2) and SSF assays (0.625 µM and 1.25 µM, Figure 3). These toxicity values were comparable to the known hepatocarcinogen AFB1, and identified CDMDC as a data-poor chemical requiring further investigation.

**Figure 4:**
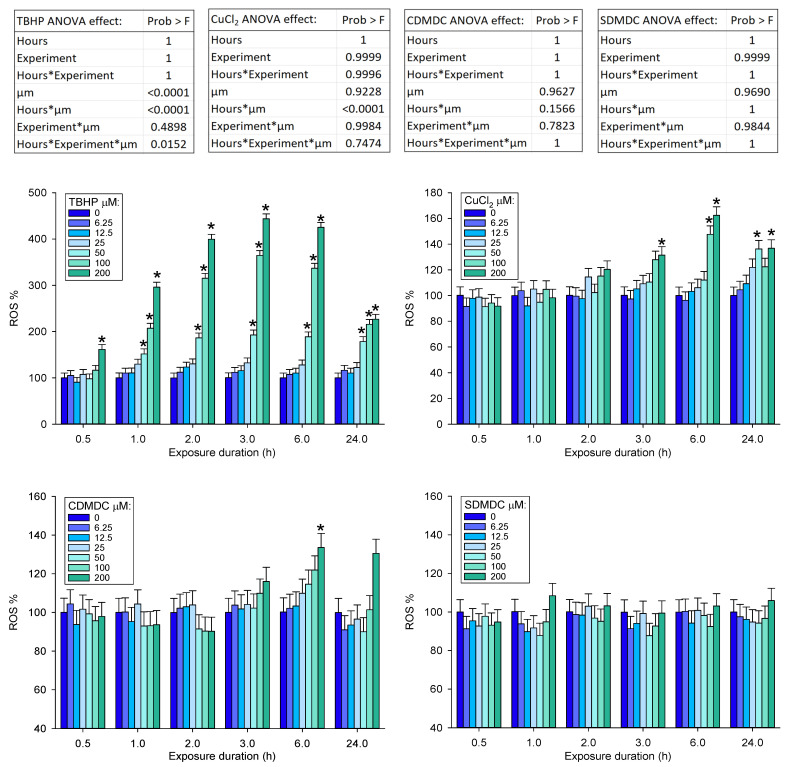
Abundance of reactive oxygen species (ROS) induced by increasing concentrations (6.25 µM to 200 µM) of copper chloride (CuCl_2_), copper dimethyldithiocarbamate (CDMDC) and sodium dimethyldithiocarbamate (SDMDC). Tert-butyl hydrogen peroxide (TBHP) was used as the positive control. The exposure time varied from 0.5 h up to 24 h. Least squares mean + standard error (SE) from a full factorial ANOVA combining three separate experiments (*n* = 3 wells/group/experiment). The CDMDC data at 6 h were also analyzed separately from other time points, testing the effects of concentration (*p* = 0.005), experiment (*p* = 0.99), and concentration X experiment (*p* = 0.99). *: indicates increases above control; Dunnett’s test *p* < 0.05).

**Figure 5:**
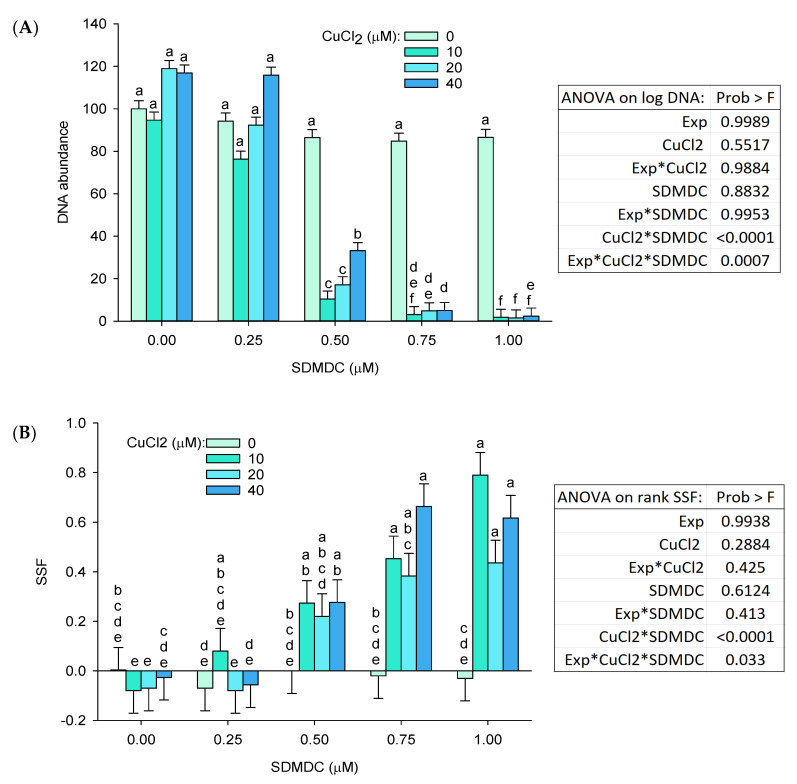

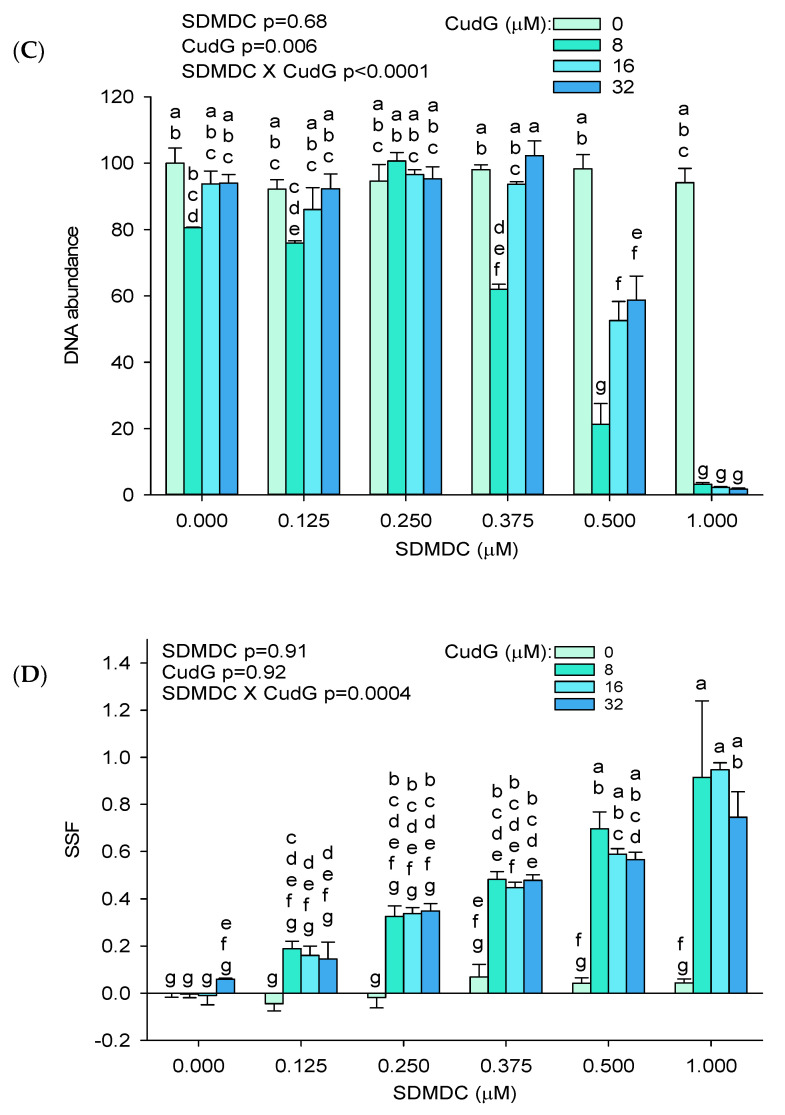
The toxicity of sodium dimethyldithiocarbamate (SDMDC) was enhanced when cells were exposed in combination with copper(II) chloride (CuCl_2_) or copper(II) D-gluconate (CuDg). Changes in DNA abundance and DNA damage expressed by the strand scission factor (SSF) following a 72 h exposure to CuCl_2_ (**A**,**B**) or to CuDg (**C**,**D**) in the presence of SDMDC. (**A**,**B**) are represented by the least squares mean + SE of a full factorial ANOVA from three separate experiments with three wells per treatment in each experiment. (**C**,**D**) are from one experiment (mean + SE, *n* = three wells per group). Means with different letters are significantly different, Tukey’s HSD *p* < 0.05. The letter “a” was assigned to the largest mean, “b” was assigned to the second largest mean, and so on. Means that share at least one letter are not significantly different.

In contrast to CDMDC, the inorganic coppers (CuCl_2_, CuBr_2_) were among the least toxic chemicals (Figure 1, Figure 2 and Figure 3). Therefore, experiments were performed to better understand the contribution of copper and of organic moiety to the toxicity of CDMDC. SDMDC was used as a surrogate for the organic moiety, whereas CuCl_2_ and CuDg were used as sources of copper. Figure 4 indicates that exposure to CuCl_2_ for only 3 h was sufficient to increase the abundance of reactive oxygen species (ROS), but six hours was required to achieve a similar response with CDMDC. In contrast, SDMDC did not induce ROS. Figure 5 monitored the induction of toxicity based on DNA abundance and DNA damage, assessed using the SSF after 72 h of exposure to mixtures of increasing concentrations of either CuCl_2_ or CuDg with/without SDMDC. The concentration ranges of CuCl_2_ or CuDg were established to cover the range of copper concentrations found in human blood (10 to 40 µM). It should be noted that while cells were exposed for 24 h in Figure 1, Figure 2 and Figure 3, they were exposed for 72 h in Figure 5. Figure 5A,B shows that, on its own, SDMDC is not toxic. Similarly, in the absence of SDMDC, increasing concentrations of CuCl_2_ or CuDg are not toxic. However, the combinations of small concentrations of copper and SDMDC reduced DNA abundance (10 µM of CuCl_2_ with 0.5 µM of SDMDC, Figure 5A; 8 µM of CuDg with 0.375 µM of SDMDC, Figure 5B). DNA damage was increased via the combination of 10 µM of CuCl_2_ with 0.75 µM of SDMDC (Figure 5C).

### 2.2. Epigenetic Effects

#### 2.2.1. Global Genome Histone Modifications

**Figure 6:**
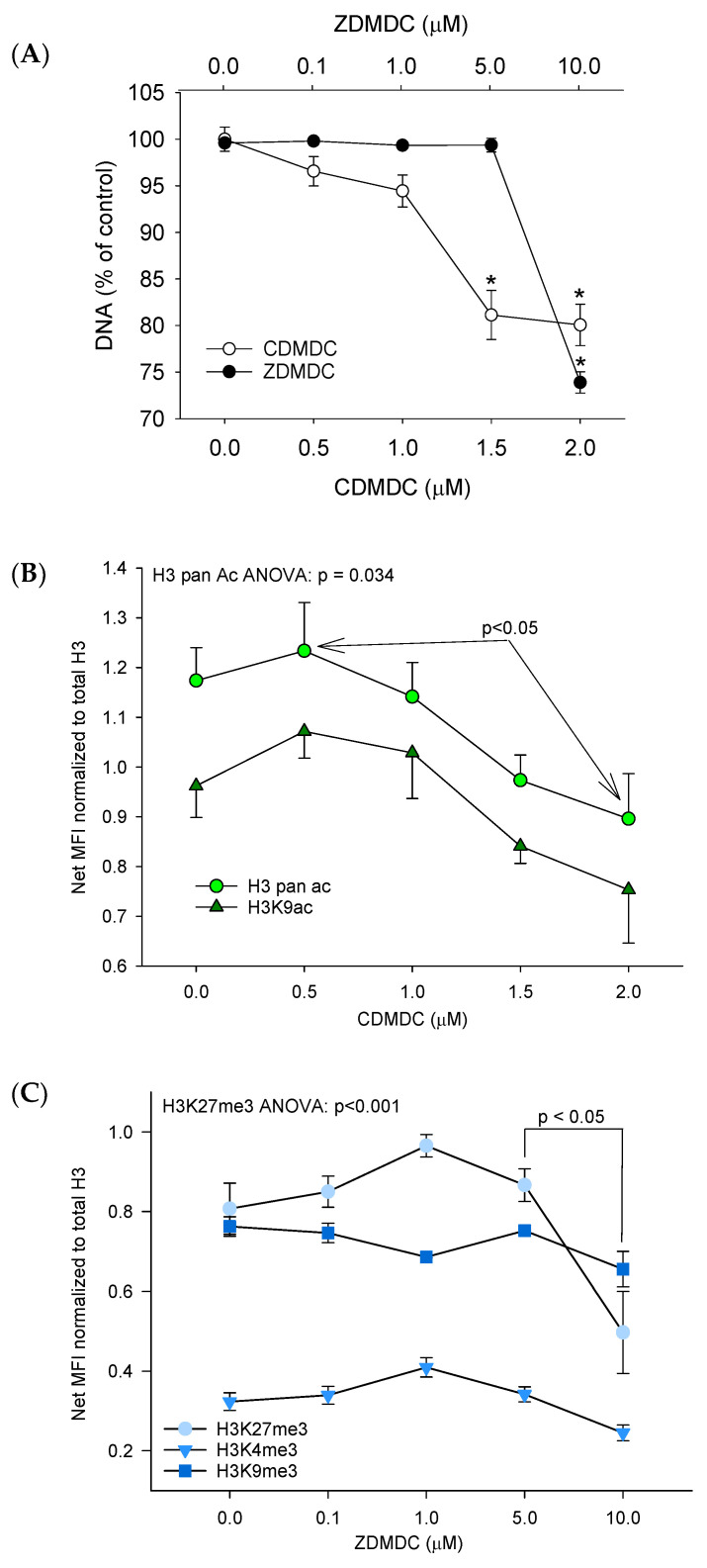
Multiplex bead array screening results for effects of 24 h exposure to copper dimethyldithiocarbamate (CDMDC) or to zinc dimethyldithiocarbamate (ZDMDC) on (**A**) the abundance of DNA relative to the control group. (**B**) CDMDC at 2 µM decreased the pan-acetylation of H3 relative to 0.5 µM (*p* = 0.03), but the effects on acetylated histone H3 lysine 9 (H3K9ac) did not reach statistical significance (error bars unidirectional to avoid overlapping). (**C**) The effects of ZDMDC on the abundance of H3K27 trimethyl (H3K27me3), H3K9me3 and H3K4me3. Mean + SE, *n* = 4. One-way ANOVA followed by Tukey’s HSD for multiple comparisons. *: different from control, *p* < 0.05. The relative histone H3 acetylation and methylation levels (**B**,**C**) are expressed as the ratio of net median fluorescence intensity (MFI) of the histone H3 modification relative to the net MFI of the total histone H3.

**Figure 7:**
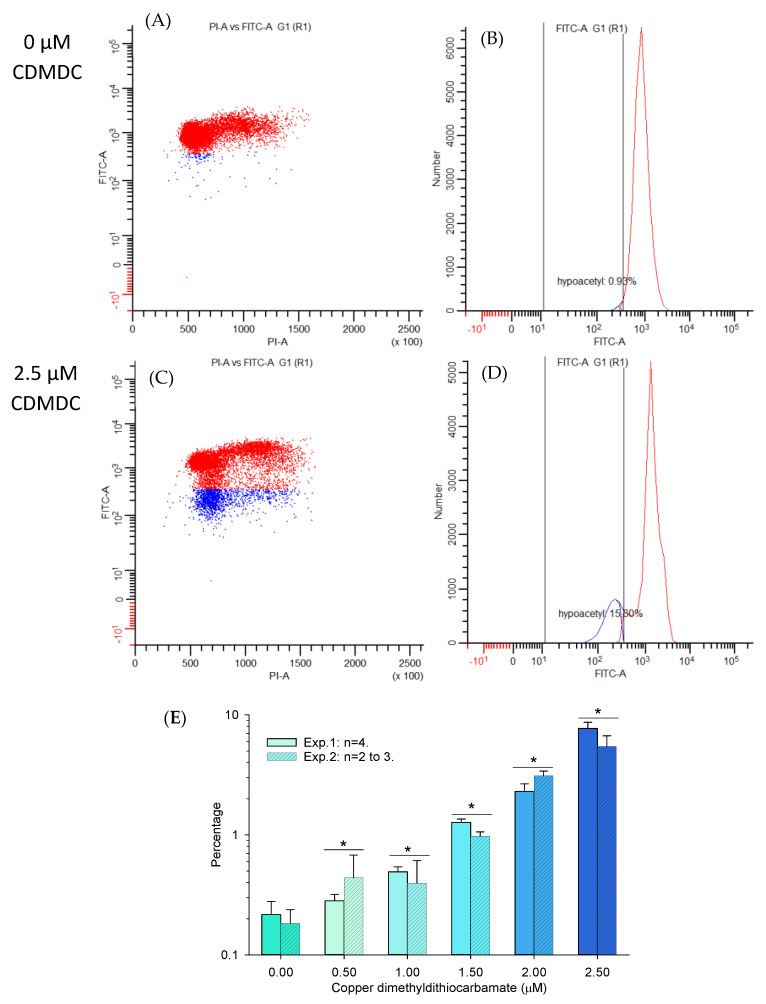
Effects of 24 h of exposure to copper dimethyldithiocarbamate (CDMDC) on histone H3 lysine 9 (H3K9) acetylation using flow cytometry and an antibody targeting H3K9Ac (FITC-A), versus genomic DNA stained with propidium iodide (PI-A). The number of hypoacetylated cells in blue was determined by a gate fixed in all samples at 2SD from the control group median. (**A**,**B**) show a control sample with a small proportion of cells being hypoacetylated in blue with reduced fluorescence intensity (Y-axis) (**B**). Exposure to 2.5 µM of CDMDC increased the percentage of hypoacetylated cells (**C**,**D**). Although a fixed gate does not permit capturing all hypoacetylated cells (**C**), this method generated a clear concentration–response pattern across two separate experiments (**E**). Mean + SE, two to four samples per group. Two-way ANOVA: concentration *p* < 0.001, experiment *p* = 0.23, interaction *p* = 0.1. *: different from control; Dunnett’s method *p* < 0.05.

The possibility of screening chemicals for concentration–response effects based on histone modifications (H3K9ac, H3-pan-ac, H3K27me3, H3K9me3 and H3K4me3) using multiplex bead arrays on a Luminex system was initially tested using two positive controls. Trichostatin-A (TSA) was used as a known histone deacetylase inhibitor; as expected, it increased H3 acetylation, but interestingly also increased the abundance of H3K4me3 (Supplementary Figure S3). GSK-126 was used as a known inhibitor of the histone methyltransferase EZH2; as expected, it reduced the abundance of H3K27me3 (Supplementary Figure S4). Then, CuCl_2_, CDMDC, ZnCl_2_, ZDMDC, ZDEDC and SDMDC were screened for the induction of histone modifications. CDMDC reduced the abundance of H3K9Ac and pan-acetyl H3 in a concentration–response manner (*p* < 0.05), from 0.5 µM up to 2 µM (Figure 6B), without any changes in the other H3 post-translational modifications (H3-PTM). ZDMDC reduced H3K27Me3 abundance (*p* = 0.005) at the highest concentration but did not affect any other H3-PTM (Figure 6C). Given that ZDMDC induced an effect only at the highest concentration, but that CDMDC decreased H3K9Ac over a wide concentration range (Figure 6), this prompted us to confirm the CDMDC observations with an alternative (cell-based) flow cytometry strategy. Figure 7 summarized two flow cytometry experiments measuring the proportion of hypoacetylated cells induced by 24 h of exposure to CDMDC. The data (Figure 7E) demonstrate a clear concentration–response pattern, with an initial increase in the proportion of hypoacetylated cells occurring at 0.5 µM of CDMDC, the lowest concentration tested here. This induction of hypoacetylation was transient and no longer detected after 72 h of exposure.

#### 2.2.2. Global Genome DNA Methylation

The methodology used to measure the global genome changes in DNA methylation was developed using the DNA methyltransferase inhibitor 5-aza-2′-deoxycytidine (5aCdR) (Figures S5 and S6), and then applied to CDMDC, CuCl_2_, SDMDC, ZnCl_2_, ZDMDC and ZDEDC.

**Figure 8:**
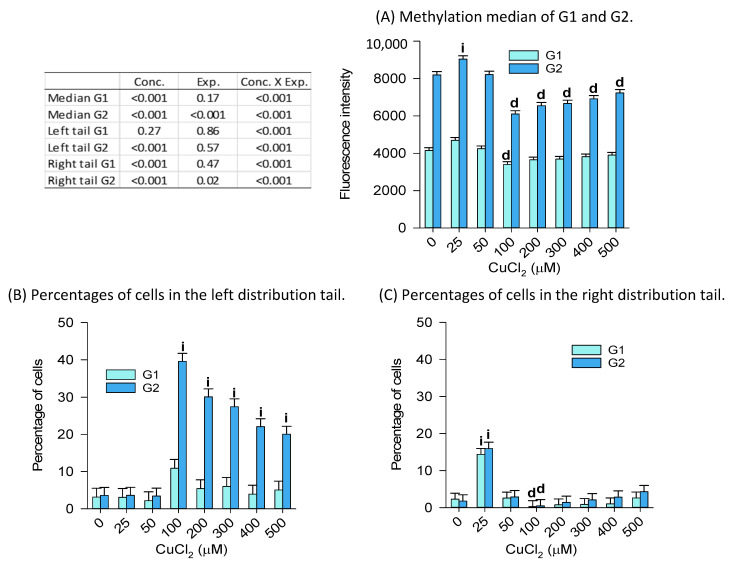
Concentration–response effects of copper chloride (CuCl_2_) on global genome DNA methylation (72 h exposure). The related percent changes in DNA methylation are presented in Figure S10. (**A**) The median fluorescence intensity of G1 cells decreased (d) significantly only at 100 µM, whereas G2 cells showed increased (i) intensity at 25 µM, followed by a reduction at concentrations equal or larger than 100 µM. This is in line with (**B**), showing the greater abundance of cells in the left tail of the G2 distribution, and (**C**), showing an increase in the percentage of cells in the right tail at 25 µM and a decrease at 100 µM. Least squares mean + SE, from three experiments with *n* = 2 to 3 samples per group within each experiment. The data in graph (**A**,**C**) were log-transformed for statistical analyses. Two-way ANOVAs followed by Dunnett’s multiple comparisons method versus 0 µM, *p* < 0.05.

**Figure 9:**
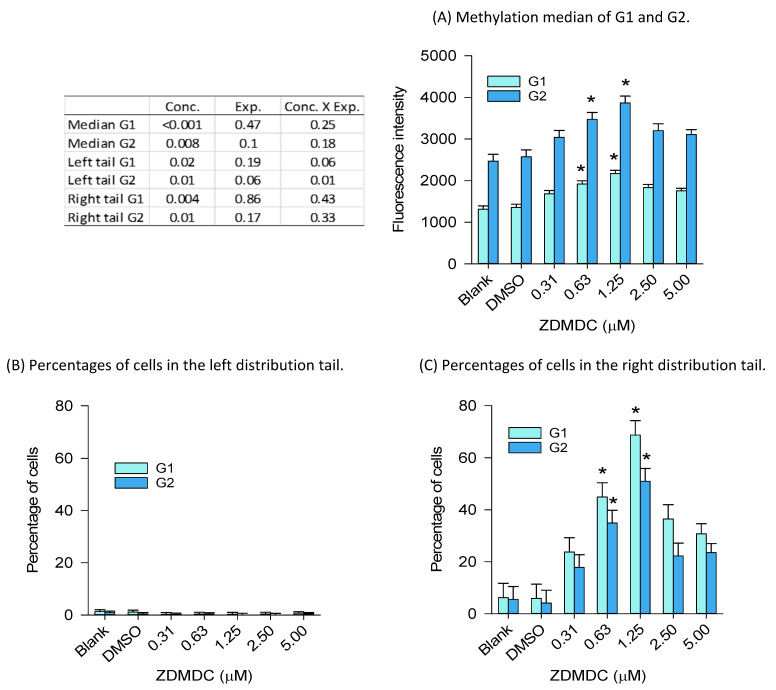
Concentration–response effects of zinc-dimethyldithiocarbamate (ZDMDC) on global genome DNA methylation (72 h exposure). The related percent changes in DNA methylation are presented in Figure S10. (**A**) The median fluorescence intensities of the G1 and G2 cell populations were significantly increased relative to DMSO, at 0.625 µM and 1.25 µM. This is consistent with (**C**), showing increases in the percentage of cells in the right tails at the same concentrations. (**B**) There was no significant effect detected in the left tails. Least squares mean + SE, from three experiments with *n* = 2 to 4 samples per group within each experiment. For (**A**–**C**), two-way ANOVA on rank followed by multiple comparisons versus DMSO; * Dunnett’s method *p* < 0.05.

In a preliminary experiment (Figure S7), the global genome DNA methylation was reduced by 24 h of exposure to 1.25 µM of CDMDC, as indicated by decreases in G1 and G2 cell fluorescence intensities and by increases in the percentages of cells in the left tail of the G1 and G2 cell populations. However, 72 h of exposure to 0.31 µM of CDMDC induced toxicity (33% of cells counted relative to DMSO), but had no effect on the global genome DNA methylation (Figure S8). These results are reminiscent of those of histone acetylation with hypoacetylation measured at 24 h, but not at 72 h. It should be noted that 24 h of exposure to CDMDC induced hypoacetylation at lower concentrations (0.5 µM, Figure 7E) than changes in the global genome DNA methylation (1.25 µM, Figure S7). Given that the heritable changes in DNA methylation across daughter cells should be more biologically relevant than early (24 h) transient changes, the next paragraph reports the global genome changes in DNA methylation after 72 h of exposure to CuCl_2_ (Figure 8) and ZDMDC (Figure 9).

The concentration–response effects of CuCl_2_ on the G1 global genome DNA methylation (Figure 8) showed a decrease in the population median only at 100 µM, whereas G2 cells showed both an increase in methylation at 25 µM (consistent with Figure 8C showing an increase in the percentage of cells in the right tail), followed by decreases in methylation from 100 µM to 500 µM. This observation is supported by the greater abundance of cells in the left tail of the G2 distribution (Figure 8B). Note that the cell number (as indicator of toxicity) declined relative to the 0 µM group (100% of cells), from 87% at 200 µM down to 67% in the 500 µM group. ). Interestingly, while copper could explain the toxicity of CDMDC (Figure 5), it appears that the DMDC moiety prevents copper having an effect on DNA methylation.

The concentration–response effects of 72 h of exposure to ZDMDC showed increases in global genome DNA methylation at 0.625 µM and 1.25 µM in both the G1 and G2 cell populations (Figure 9). These effects were associated with increases in the percentage of cells in the right tails occurring at the same concentrations. The cell number declined relative to the DMSO group (100% of cells), from 75% at 0.625 µM, 69% at 1.25 µM, down to 65% in the 5 µM group.

Overall, it appears that the induction of DNA methylation changes at 72 h is a chemical-specific effect occurring at concentrations close to those associated with a reduction in cell numbers and the emergence of toxicity.

## 3. Discussion

The “data-poor” copper and zinc organometallic screening data (24 h exposure) on DNA abundance, the induction of the pH2AX/DNA ratio, and SSF suggest a toxicity hierarchy starting with CDMDC > ZDEDC > BDCN4CZ. Subsequent positions on this toxicity scale alternate between different chemicals depending on the assays. The further investigation of copper, SDMDC and CDMDC suggested the interaction between copper and the DMDC organic moiety, resulting in higher-than-expected CDMDC toxicity. In contrast, such effects were not observed in DNA methylation flow cytometry data. The latter revealed chemical-specific effects with hypo and hypermethylation induced by 72 h of exposure to CuCl_2_ and ZDMDC, respectively, but did not show the significant effects of CDMDC or SDMDC treatment. Histone-3 hypoacetylation was a sensitive flow cytometry marker of 24 h of exposure to CDMDC. CDMDC was shown to induce DNA damage with a potency close to that of the human hepatocarcinogen AFB1, which is of consideration in chemical hazard assessment.

The high toxicity of CDMDC prompted us to further investigate the contribution of the copper and organic DMDC moieties to the toxicity of CDMDC. This was achieved by assessing the effects of SDMDC over a range of Cu^2+^ concentrations that exist in human blood (using CuCl_2_ or CuDg as sources of Cu^2+^). The reference range for copper in human serum was reported as 12.0–20.0 µM [31]. However, some individuals have concentrations that exceed 40 µM [31,32]. Therefore, concentrations of CuCl_2_ or CuDg up to 40 µM were tested against up to 1 µM of SDMDC. Interestingly, while both forms of copper (CuCl_2_, CuDg) and SDMDC had low toxicity when tested separately, the combination of ≥0.375 µM of SDMDC with ≥8 µM of Cu^2+^ showed interactions resulting in significant toxicity (Figure 5C). A similar pattern was observed by others testing the effects of a different metal chelator, the aminothiol *_D-_*penicillamine [reviewed in [33]]. Neither *_D-_*penicillamine nor copper alone caused cytotoxicity in human cancer cells, but when both were incubated together, copper catalyzed *_D-_*penicillamine oxidation and the subsequent formation of H_2_O_2_ and cytotoxicity in endothelial cells, lymphocytes and leukemia cells [33]. Perhaps the same phenomenon might be at play for CDMDC, as copper can oscillate between the cupric Cu^2+^ and cuprous Cu^+^ cations in the presence of O_2_ and a reducing agent, whereby the sulfhydryl group of the DTC reduces Cu^2+^ and induces oxidative stress reactions [33,34]. The relative toxicity of CDMDC > ZDMDC > ZDEDC observed here might be linked to the fact that, in contrast to copper, zinc is a redox-inactive metal [35]. The toxicity data reported here are further supported by the medical application of analogous DTCs with copper supplementation as anti-cancer therapy [5,36]. Differences in toxicity, based on the molar ratio of copper and DMDC in a mixture, might impact on the design of biocide mixtures and chemical safety regulation.

It is interesting to note that in the presence of an organic moiety (DMDC), copper in CDMDC was more toxic than zinc in ZDMDC; meanwhile, in an inorganic context, the relative toxicity of ZnCl_2_ was greater than CuCl_2_ and CuDg. The lower toxicity of inorganic copper compared to zinc might be associated with the function of the hepatocytes and the greater homeostasis potential (intake, cellular compartmentalization, efflux) for copper [24,33] than zinc [37,38,39]. Both copper and zinc are essential trace elements and components of numerous proteins and enzymes. Zinc is involved in numerous protective cellular functions (e.g., oxidative stress, DNA damage response, cell proliferation/apoptosis, zinc-finger transcription factors, epigenetic enzymes). At low concentrations, it can induce proliferation and act as a transcriptional regulator and second messenger, transducing extracellular stimuli into intracellular signaling events [40,41]. At concentrations exceeding 170 µM, zinc induces cytotoxicity in HepG2 cells [42], which is similar to the concentration–response toxicity results observed here for ZnCl_2_. 

DTCs induce toxicity through numerous mechanisms. They are metal chelators reported to be mostly neurotoxic and hepatotoxic based on the catabolic production of the neurotoxic carbon disulfide, which leads to the transformation of ethylenethiourea and the inhibition of cholinesterases, and the production of carcinogenic nitrosamine precursors [3,43]. Diethyldithiocarbamate inhibits Cu/Zn-SOD as well as Fe and MnSOD [44]. Epigenetic effects can be added to this list of mechanisms. The current flow cytometry analyses demonstrated transient (24 h) CDMDC-induced histone hypoacetylation. These results are supported by histone hypoacetylation induced by other DTCs analogues, which in the presence of Cu^2+^, inhibit histone acetyltransferase but not histone deacetylase [26]. Histone hypoacetylation is linked to chromatin condensation, transcriptional silencing, DNA repair, and cell cycle checkpoint recovery [45,46]. Histone acetylation is becoming more frequently investigated in toxicity studies (e.g., arsenic trioxide [47], ultraviolet radiation [48], formaldehyde [49]). In 72 h experiments, CuCl_2_-induced DNA hypomethylation might be linked to the CuCl_2_ induction of oxidative stress reported herein. Oxidative stress induces the formation of 8-oxo-7,8-dihydroguanosine (8-oxo-dG), which is recognized by 8-oxo-dG DNA glycosylase (OGG1) to initiate DNA repair and to stimulate demethylation of cytosines adjacent to 8-oxo-dG [50,51]. Zhou et al. [52] indicated that OGG1-bound 8-oxo-dG recruits the DNA demethylase TET1, which then initiates cytosine demethylation. Moreover, 8-oxo-dG weakens DNMT3a/DNA bonding, consequently reducing DNA methylation [53,54]. ZDMDC induces bimodal effects with DNA hyper and hypomethylation at low and high concentrations, respectively. Such bimodal effects might be linked to the ZnCl_2_ bimodal response discussed in the previous paragraph. Other DTCs were reported to affect the epigenetic system, such as disulfiram, which is a drug used to treat alcohol dependence [55,56,57]. Disulfiram induces DNA hypomethylation [58], but also inhibits the histone lysine demethylase 4A (that demethylates H3K9me3) by ejecting the zinc ion from a structural site of the demethylase [59]. Interestingly, the abundance of ethylene thiourea in boys’ spot urine samples, a metabolite of ethylene-bis-dithiocarbamate fungicides currently in use, is linked, alongside other chemical metabolites, to higher DNA methylation and a lower expression of the gene brain-derived neurotrophic factor (BDNF; a proposed biomarker of neurological function, [60]). Such observations raise concerns about contemporary non-persistent biocides and the ZDMDC induction of DNA hypermethylation observed here at low concentrations. 

Prior to initiating epigenetic investigations demanding expensive next-generation sequencing and bioinformaticians to describe gene and site-specific epigenetic modifications across the genome, the use of flow cytometry is an efficient screening approach used to identify chemicals for global genome changes in DNA methylation and histone modifications. Determining the percentage of G1 and G2 cells affected through population distribution analyses was a sensitive approach to establishing the concentration–response patterns that are necessary in chemical hazard assessment. However, a further understanding of the consequences of these global epigenetic changes is needed to strengthen their consideration regarding mechanisms of action in chemical hazard assessment.

## 4. Materials and Methods

### 4.1. Chemicals

Table 1 provides a list of the copper and zinc organometallic chemicals and positive control chemicals with their suppliers and CAS numbers. It also indicates whether the chemicals were diluted in water or in a final dilution of 0.05% dimethylsulfoxide (DMSO; Sigma-Aldrich, Oakville, ON, Canada). The dilutions of CDMDC were unstable and were prepared fresh for each experiment. The structures of the dithiocarbamate chemicals are described in [1]. 4-nitroquinoline-N1-oxide (NQO) and hydrogen peroxide (H_2_O_2_) were selected as positive controls because they do not need to be transformed by cytochrome p450 (CYP) enzymes to become reactive to DNA and induce DNA single-strand breaks (SSB). In contrast, AFB1 must be transformed to become reactive to DNA and to induce double-strand breaks (DSB); it was selected as a third positive control. The positive controls for epigenetic analyses included the histone deacetylase inhibitor trichostatin-A, the histone methylase inhibitor GSK126, and the DNA methyltransferase inhibitor 5aCdR (Decitabine), all from Sigma-Aldrich and tested in a dilution of 0.5% DMSO.

### 4.2. HC-04 Cell Line and Culture Conditions

The HC-04 human cell line is a spontaneously immortalized hepatocyte cell line that was established using liver tissues surrounding a hepatoma in a male patient. It was obtained from the Biodefense and Emerging Infections Research Resources Repository (Manassas, VA, USA). Morphologically, HC-04 cells resemble liver parenchymal cells and proliferate with a doubling time of approximately 24 h. A cytogenetic analysis revealed that they exhibited a hyperdiploid karyotype (range 47–50), with consistent abnormality on chromosome 1 [t(1;21)], 6 [del(6q)], and 15 [8der(15)] [61]. The HC-04 cell line was reported to have maintained numerous inducible Phase-I, -II, and III enzymes [62]. Here, a preliminary experiment suggested that the CYP1A1 and CYP3A4 activities were greater in HC-04 than in HepG2 cells (Supplementary Figure S1). As some organometallic compounds may need to be transformed by the CYP system to become reactive to DNA, the HC-04 cell line was selected for this project. Experiments were conducted with HC-04 cells from passages 56 to 69.

Hepatocyte Culture Medium (HCM) was formulated using HBM Basal Medium (CC-3199) and HCM SingleQuots^TM^ supplements (CC-4182) obtained from Lonza (Walkersville, MD, USA). SingleQuots^TM^ provided the hepatocyte growth and other factors, including ascorbic acid, bovine serum albumin (BSA)-fatty acid free, hydrocortisone, transferrin, insulin, rhEGF (recombinant human epidermal growth factor), gentamicin sulfate, and amphothericin-B, at proprietary concentrations. The fibronectin, BSA fraction V, collagen I, and DMSO (sterile tissue culture grade) were from Sigma-Aldrich, whereas the trypsin/EDTA (0.25%), fetal bovine serum (FBS), and Dulbecco’s modified phosphate-buffered saline without magnesium or calcium (D-PBS) were from Gibco/Invitrogen (Burlington, ON, Canada). The corning cell culture dishes, which were 100 mm and 96-well black culture plates for fluorescence, were from VWR (Mississauga, ON, Canada).

The HC-04 cells were grown in pre-coated culture dishes prepared by incubating the flasks for 2h (37 °C, 5% CO_2_) with HBM containing BSA, fibronectin (0.01 mg/mL each), and collagen (0.03 mg/mL). The coating media was removed, and the dishes were rinsed with D-PBS prior to seeding. The HC-04 cells were maintained in HCM medium supplemented with 10% FBS in 100 mm culture dishes at 37 °C, 5% CO_2_, 100% humidity, and the media was replaced every three to four days. When the cells reached approximately 80% confluency, the plates were washed once with D-PBS and then the cells were detached via treatment with trypsin/EDTA for 5 to 10 min at 37 °C. Trypsinization was stopped with complete medium, then cells were collected via centrifugation at 4 °C, washed, and reseeded in a 1:3 split ratio, or used in specific experiments.

### 4.3. DNA Damage Assays

#### 4.3.1. γH2AX Assay

The measurement of pH2AX provides an indication of the functionality of enzymatic systems regarding the detection and signaling of the presence of DNA damage. DNA DSBs activate the ataxia-telangiectasia mutated (ATM) and DNA-PKcs (DNA-dependent protein kinase catalytic subunit), which phosphorylate multiple DNA repair proteins, including histone H2AX; these then become binding sites for DNA repair proteins. Phosphorylated H2AX provides a binding site for MDC1 (Mediator of DNA Damage Checkpoint 1), which promotes the spreading of pH2AX (then called gamma-H2AX (γH2AX)) for hundreds of kilobases on either side of the break [63,64].

The abundance of pH2AX was measured using the commercial ELISA assay kit “Human/Mouse/Rat Phospho-Histone H2AX (S139) Immunoassay” using the reagents and instructions provided by the manufacturer (R&D systems, Minneapolis, MN, USA). H2AX is partly linked to DNA synthesis; therefore, elevated H2AX phosphorylation might partially result from activated DNA synthesis [65,66]. Therefore, the current assay reports the abundance of pH2AX as the ratio pH2AX/DNA. Methodological details are provided in the Supplementary Materials.

#### 4.3.2. DNA Alkaline Unwinding Assay

Multiple DNA unwinding-based assays were compared [67,68,69,70] and finally the Fast Micromethod [71,72,73] was selected for its simplicity. However, one modification included the addition of 0.05% Triton X-100 (Sigma-Aldrich) to the dye-binding buffer to improve the penetration of the dye into cells. This method relies on two principles: the first is that exposure to alkaline conditions causes double-stranded DNA to unwind at a lower rate than damaged DNA, and the second is that the PicoGreen DNA dye (Life Technologies, Burlington, ON, Canada) emits fluorescence only when bound to double-stranded DNA but not single-stranded DNA or proteins. Thus, after exposing cells to PicoGreen then measuring the drop in fluorescence due to the release of PicoGreen during DNA unwinding under alkaline conditions, an index of DNA damage is calculated and referred to as the SSF. The SSF is calculated at a specific time after the addition of the alkaline solution that initiates DNA unwinding. The detailed methodology and SSF formula are presented in the Supplementary Materials.

### 4.4. Reactive Oxygen Species Assay

ROS generation was assessed using the 2’,7’-diclorodihydrofluorescein di-acetate (DCFDA) fluorescence assay (Abcam Inc. Toronto, ON, Canada). This assay measures hydroxyl, peroxyl and other ROS activity within the cell, and was performed as described by the manufacturer. Black, clear-bottom 96-well culture plates were coated with collagen, as previously described, and seeded overnight with 25,000 cells per well. The medium was aspirated; cells were washed with 1× buffer (provided with the kit) and then incubated with the diluted DCFDA solution for 45 min at 37 °C in the dark. At the end of this period, the DCFDA solution was removed and replaced with HC-04 cell medium containing the test chemicals at concentrations ranging from 6.25 to 200 µm and exposed for 0.5, 1, 2, 3, 4, 5 and 6 h. TBHP (tert-butyl hydrogen peroxide) was used as the positive control. The fluorescence intensity was measured for each period at wavelengths of 485 nm (excitation) and 527 nm (emission) on a microplate reader (Molecular devices Spectra Max M2, Sunnyvale, CA, USA). The data were expressed as percentage of the vehicle controls (water for CuCl_2_ and SDMDC, and 0.5% DMSO for CDMDC). All experiments were repeated three times on different days.

### 4.5. Epigenetic Assays 

#### 4.5.1. Screening for Histone Post-Translational Modifications Using Luminex Multiplex Bead Array

To extract the total histones, HC-04 cells (2 to 5 × 10^5^) were trypsinized and then collected in microcentrifuge tubes, washed twice with D-PBS via successive suspension and centrifugation at 200× *g*, then frozen at −30 °C until lysis. To deplete the non-histone proteins, the cell pellets were suspended via vortex in cold PBS-TrA (D-PBS with 1% BSA and 0.1% Triton X-100), incubated on ice for 10 min, and then the nucleus-enriched fraction was precipitated at 10,000× *g* for 10 min at 4 °C. The pellet was suspended via pipetting with 30 µL of 0.4 M hydrochloric acid then incubated with agitation for one hour at 4 °C. The histone suspension was clarified via centrifugation at 10,000× *g* for 10 min at 4 °C; then, the supernatant was transferred to a new tube. The pH of the supernatant was neutralized by adding a 40% volume of neutralization buffer (0.5 M sodium phosphate tribasic, pH 12.5) containing 2.5 mM of dithiothreitol (Sigma-Aldrich) and 3.5% by volume of each proprietary enzyme (protease, deacetylase, and phosphatase) inhibitor supplied by Active Motif (Carlsbad, CA, USA). The protein concentration of the histone extracts was estimated using the Bradford method [74] with BSA protein standards. The protein concentration of each sample was normalized to the least concentrated sample using the equivalent solution of hydrochloric acid/neutralization buffer/inhibitors.

Components from a Histone H3 Post-Translational Modification (PTM) Multiplex Kit from Active Motif were used in the multiplex bead array. A set of six fluorescence-labelled magnetic beads conjugated to antibodies targeting five histone H3 post-translational modifications (H3K9Ac, H3pan-acetyl, H3K4Me3, H3K9Me3, and H3K27Me3) and one total histone H3 (primary sequence epitope) antibody were mixed with the kit-supplied binding buffer (AM3) and 750 ng of histone extract; this mixture was then incubated in white, opaque, round-bottomed 96-well Costar plates (#3789, Corning Life Sciences, Tewksbury, MA, USA), with shaking at 700 rpm for 1 h. The beads were washed twice by first capturing them on a Lifesep magnetic plate (Dexter Magnetic Technologies, Elk Grove Village, IL, USA), inverting the plate to discard the solution, and then suspending the beads in the kit-supplied 1× wash buffer. The beads were then similarly incubated with biotin-conjugated histone H3 antibody for one hour, followed by Streptavidin-PE for 30 min with 2 washes between each incubation. The median fluorescence intensity (MFI) of each bead type in each sample was measured using the Luminex^®^ 200^TM^ system (Luminex, Austin, TX, USA). The degree of histone H3 modification was represented as the Net MFI ratio from H3 PTM versus the total H3.

#### 4.5.2. Flow Cytometry Analyses for the Quantification of H3K9 Acetylation

Adherent HC-04 cells were washed once with D-PBS then detached via treatment with trypsin/EDTA for 5 to 10 min at 37 °C. Trypsinization was stopped with ice-cold complete medium; then, the cells were collected via centrifugation at 4 °C. All further washes were carried out in ice-cold solutions.

Cells were permeabilized in PBS-Tw (D-PBS with 1% BSA and 0.1% Tween20 (Sigma-Aldrich)) for 10 min then pre-fixed in formaldehyde (Life Technologies) for 15 min. Ethanol fixation alone is not compatible with acetyl histone H3 detection [75,76]. Histone acetylation was more sensitively detected in cells fixed with 4% formaldehyde; this was a concentration that impaired cell cycle analysis. Better-resolved G1/G0 and G2/M peaks were obtained with 1% (or 0%) formaldehyde fixation, but the histone acetylation could not be sensitively detected. Therefore, a formaldehyde concentration of 2% was chosen as a trade-off to optimize both the acetylation detection sensitivity and cell cycle resolution.

After formaldehyde fixation, the cells were washed once with D-PBS, fixed in 70% ethanol, and then stored at −30 °C until staining. Fixed cells were rehydrated in D-PBS, then blocked for 10 min with PBS-TrA, and then stained overnight at 4 °C in the dark with a 1:50 dilution (in PBS-TrA) of histone H3K9-acetyl antibody (C5B11) Alexa Fluor^®^ 488 conjugate (New England Biolabs, Whitby, ON, Canada). Cells were washed 1× with PBS-TrA before suspension in 1 mL of D-PBS with 5 µg/mL of propidium iodide (PI; Life Technologies) and 40 µg/mL of RNAse A (Life Technologies); this was then filtered through strainer caps into tubes (BD, Heidelberg, Germany). Flow cytometry was performed with an LSRII (BD) instrument, which detected Alexa Fluor^®^ 488 using a 488 nm laser excitation with a 525/50 bandpass emission filter and detected PI using a 561 nm laser with a 610/620 filter. Since single cells in G2 can be masked by G1 doublets, Flowjo software v7.6 (Tree Star, OR, USA) was used to plot the PI area versus PI width to gate singlet cells for further cell cycle and histone acetylation analysis [77].

#### 4.5.3. DNA Methylation by Flow Cytometry

This protocol describes the sample preparation steps, including cell fixation, cell permeabilization to facilitate staining with primary and secondary antibodies, and then propidium iodide staining. HC-04 cells were seeded into 6-well plates (at 3 × 10^5^ or 1.5 × 10^5^ cells in 24 h or 72 h exposure experiments, respectively) and incubated overnight. The cells were then exposed to complete medium containing test chemicals for 24 or 72 h, then rinsed with 1× D-PBS and harvested via trypsinization using a 2:1 ratio mixture of trypsin/EDTA. Detached cells were suspended in culture medium and were pipetted up and down to dissociate any clumps; they were then counted to assess cytotoxicity. Harvested cells were washed with 5 mL of PBS-Tw, pelleted (centrifuged for 4 min at 300× g), and then fixed with 70% ethanol at −20 °C. Fixed cells were permeabilized with PBS-TrB (D-PBS, 0.05% Triton X-100, 1% BSA) at room temperature (RT) for 5 min and pelleted. The cell pellets were resuspended with 1 mL of 2N HCL for 40 min at RT, washed in 4 mL of 1× D-PBS, pelleted, neutralized via resuspension in 3 mL of 0.2 M phosphate buffer at pH 7.4, pelleted and resuspended in 1 mL of PBS-TrB for cell counting (Scepter Cell Counter, EMD Millipore, Etobicoke, ON, Canada). This cell count was necessary to select a volume that could generate cell pellets with similar cell numbers (2.5–3 × 10^5^ cells) across all samples to ensure that the abundance of cells relative to that of the primary antibody remained constant across experimental groups. Then, 150 µL of PBS-TrB with 0.5 µg of primary antibody (monoclonal anti-5-methylcytosine antibody, clone 162 33D3, cat# NA81, EMD Millipore) was added into each sample and incubated with gentle agitation at RT for 1 h. The cells were washed with PBS-TrB, resuspended in 150 µL of PBS-TrB with 6 µg of goat anti-mouse secondary antibodies coupled with ALEXA FLUOR^®^ 488 (Life Technologies), and incubated in darkness with gentle agitation at RT for 45–60 min. The cells were PBS-TrB-washed, pelleted, and stained for DNA by resuspending them in 500 µL of D-PBS with 5 µg/mL of propidium iodide (PI; Life Technologies) and 50 µg/mL of RNase (Life Technologies). Finally, the cells were filtered using a tube with a cell-strainer cap (BD Falcon, Fisher Scientific, Ottawa, ON, Canada) before being examined via flow cytometry (Beckman Labcell Quanta, Beckman Coulter Canada, Mississauga, ON, Canada), using appropriate wavelength settings: 488 nm excitation, PI emission at >620 nm, ALEXA FLUOR^®^ 488 emission at 530 ± 20 nm. The flow cytometry data were derived from the analyses of 20,000 cells.

The global genome changes in DNA methylation induced via 24 h of incubation with the DNA methyltransferase inhibitor 5aCdR(0 µM, 5 µM and 50 µM) were used to develop the flow cytometry methodology (Figures S5 and S6). Data were analyzed using the software WinList v.8.0 (Cytonome Verity, Bedford, MA, USA), ModFit LT v.4.0 (Cytonome Verity) and Flowjo v7.6 (BD Life Sciences, Ashland, OR, USA). A fixed-pulse geometry gate (X:FSC-A, Y:FSC-H) was created based on the control samples to exclude small and large particles. Using the graph with FITC-A (DNA methylation) on the X-axis and PI-A (DNA abundance) on the Y-axis, ellipses were used to gate the G1 (diploid) and G2 cells (tetraploid), which permitted us to investigate these cell populations separately across the cell cycle (Figure S5A,C,E). Given that the G1 and G2 cell populations were investigated separately, each sample generated three endpoints for the G1 and three others for the G2 cell populations. These were the median fluorescence signal for each cell population, and the proportion of cells in the left (hypomethylation) and right tails (hypermethylation) of the distribution within each sample (Figure S5B,D,F; examples of G1 only). Changes in the medians across treatment groups reflect shifts in the entire cell populations and are thus strong indicators of DNA methylation changes, whereas tail distributions can be more sensitive, indicating effects in the small proportion of cells undetected in median analyses. The gates to define the limits for the lower and upper tails were based on the distributions of the control samples and then fixed for treatment groups. Briefly, the samples of the control group were used to calculate the average distribution median and the average of the standard deviations (SD); then, the gate limits were fixed at the average median, plus or minus two SD, as shown on the frequency distribution graphs (G1 cell population distributions in Figure S5B,D,F). As expected, 5aCdR induced global genome DNA hypomethylation in a concentration–response manner, with a decrease in the population median fluorescence intensity, as indicated by the movement of the population toward the left side; this is associated with increases in the percentages of cells in the left tail (hypomethylation) of both the G1 and G2 cells (G1 cells from 1.4% to 49%; Figure S5B,F). The graphs also showed that not all cells responded equally to the treatment, creating a separation among the G2 and G1 cells (Figure S5E); this was with the identification of two G1 cell populations using the frequency distribution graphs (Figure S5F).

### 4.6. Data Analyses

The software SigmaPlot v13.0.0.83 (Systat Software Inc., Knoxville, TN, USA) and JMP v 14.1.0 (SAS Institute Inc., Cary, NC, USA) were used to analyze the data. Normality (Shapiro–Wilk test) and the equality of variances (Brown–Forsythe test) were confirmed prior to performing one-way or two-way analyses of variance (ANOVA). Then, the smallest concentration inducing a statistically significant response different from the control was identified using multiple comparison tests. If the normality or equality of variance tests failed, the data were log-transformed and retested for normality and equality of variances. If the log-transformed data failed the normality and equality of variances tests, then non-parametric ANOVA tests on ranks were used, followed by the Dunn’s method for multiple comparisons versus the control group [78]. Instead of presenting one typical experiment when multiple experiments were repeated, an effect of experiment was added to the ANOVA; the data were presented using the least squares means and standard errors (SE) to summarize the data from all experiments in a single graph. In all cases, *p* < 0.05 indicated a significant difference.

## 5. Conclusions

The comparison of nine Cu and Zn inorganic and organometallic “data-poor” chemicals with structurally related chemicals and positive controls revealed that the toxicity of organic molecules can be greatly modified via combination with metals, such as zinc or copper. The toxicity of the DTCs was enhanced by the addition of a copper or zinc moiety, while the toxicity of phenolsulfonate and toluenesulfonate was potentiated by a zinc moiety. The induction of DNA damage via CDMDC was close to that of the hepatocarcinogen AFB1, and the induction of cellular proliferation and DNA damage caused by ZnCl_2_ all raise concerns regarding the potential fixation of DNA damage into mutations and thus the initiation of carcinogenic events. The increased toxicity of DTC compounds caused by copper, and the potential for environmental exposure to both chemicals [3], may raise risk assessment concerns. To identify reliable DNA methylation and histone modification markers of prognostic value in chemical hazard assessments, further research is needed (notably on the magnitude of the response to reach adversity, the genomic site and chemical specificity, mechanisms, differences across cell types and individual variability) to improve our understanding of the consequences and of their chronology with regard to the sequence of events associated with carcinogenesis. Overall, this study identified CDMDC as the most toxic of the “data-poor” Cu and Zn organometallics tested here, and provides insights regarding chemicals and the prioritization of studies, with the aim being to mitigate chemical hazards.

## Figures and Tables

**Table 1:** List of inorganic and organometallic copper and zinc chemicals. Here, the term “organometallic” is also used for chemicals that are coordination compounds with no metal–carbon bond.

Name	Supplier	CAS	Abbreviation	Water Soluble
**Inorganic copper and zinc chemicals**				
Copper(II) chloride dihydrate	Sigma-Aldrich, Oakville, ON, Canada	10125-13-0	CuCl_2_*	Yes
Copper(II) bromide	Sigma-Aldrich	7789-45-9	CuBr_2_*	Yes
Zinc Chloride ^a^	Sigma-Aldrich	7646-85-7	ZnCl_2_	Yes
**Organic chemicals**				
Dietary supplement				
Copper(II) D-gluconate	Sigma-Aldrich	527-09-3	CuDg*	Yes
**Rubber vulcanization, fungicide wood preservative (dithiocarbamate related, they can form nitrosamines)**				
Copper(II) Dimethyldithiocarbamate	Spectrum Chemical MFG Corp, New Brunswick, NJ, USA, and Tokyo Chemical Industry (TCI), Cambridge, MA, USA	137-29-1	CDMDC*	0.05% DMSO
Zinc diethyldithiocarbamate	Sigma-Aldrich	14324-55-1	ZDEDC*	0.05% DMSO
Zinc dimethyldithiocarbamate (Ziram) ^a^	Sigma-Aldrich	137-30-4	ZDMDC	0.05% DMSO
Sodium Dimethyldithiocarbamate (Dibam) ^a^	Sigma-Aldrich	128-04-1	SDMDC	Yes
**Dyes (benzenediazonium related)**				
Benzenediazonium,3-methyl-4-(1-pyrrolielinyl)-,Trichlorozincate hydrate	Waterstone Technology Carmel, IN, USA	52572-38-0	BDMP3CZ*	Yes
Benzenediazonium, 4-chloro-2-nitro-, tetrachlorozincate(2-) (2:1). Fast Red 3GL salt	Molekula Irvine, CA, USA	14263-89-9	BDCN4CZ*	Yes
Sudan I, 1-Phenylazo-2-naphthol ^a^	Sigma-Aldrich	842-07-9	Sudan	0.05% DMSO
**Mouthwash, biocide, soap**				
Zinc Phenolsulfonate	Spectrum Chemical MFG Corp, New Brunswick, NJ, USA	127-82-2	ZPS*	Yes
Phenolsulfonic acid ^a^	Sigma-Aldrich	98-67-9	PSA	Yes
Zinc p-toluenesulfonate hydrate	Sigma-Aldrich	13438-45-4	ZpTS*	Yes
Sodium p-toluenesulfonate ^a^	Sigma-Aldrich	657-84-01	SpTS	Yes
Benzoic acid, 4-(1,1-dimethylethyl)-, zinc salt (2:1)	BOC Sciences, Shirley, NY, USA	4980-54-5	BADZ*	0.05% DMSO
**Positive controls**				
Aflatoxin-B1	Sigma-Aldrich	1162-65-8	AFB1	0.05% DMSO
4-nitroquinoline-oxide	Sigma-Aldrich	56-57-5	NQO	0.05% DMSO
Hydrogen peroxide	Sigma-Aldrich	7722-84-1	H2O2	Yes
Trichostatin-A	Sigma-Aldrich	58880-19-6	TSA	0.5% DMSO
GSK126	Sigma-Aldrich	1346574-57-9	GSK126	0.5% DMSO
5-aza-2′-deoxycytidine	Sigma-Aldrich	2353-33-5	5aCdR	0.5% DMSO

^a^: These chemicals were added as structurally related chemicals; they are not “data-poor” chemicals identified by an asterisk beside the abbreviated names.

## Data Availability

Data presented in this study are available upon request from the corresponding author. The data are not publicly available due to the large number of files involved.

## Supplementary Materials

The supporting information can be downloaded at: https://www.mdpi.com/article/10.3390/ijms242115580/s1. References [79,80,81] are cited in the Supplementary Materials.

## Abbreviations

**5aCdR**	5-aza-2’-deoxycytidine.
**AFB1**	aflatoxin-B1.
**BADZ**	benzoic acid: 4-(1,1-dimethylethyl)-, zinc salt (2:1)*.
**BDCN4CZ**	benzenediazonium, 4-chloro-2-nitro-, tetrachlorozincate(2-) (2:1). Fast Red 3GL salt*.
**BDMP3CZ**	benzenediazonium,3-methyl-4-(1-pyrrolielinyl)-,trichlorozincate hydrate*.
**BER**	base excision repair.
**CDMDC**	copper(II) dimethyldithiocarbamate*.
**CuBr_2_**	copper(II) bromide*.
**CuCl_2_**	copper(II) chloride dehydrate*.
**CuDg**	copper(II) D-gluconate*.
**CYP**	cytochrome p450.
**DTC**	dithiocarbamate.
**DSB**	DNA double-strand breaks.
**NER**	nucleotide excision repair.
**NQO**	4-nitroquinoline-oxide.
**PSA**	phenolsulfonic acid.
**ROS**	reactive oxygen species.
**SDMDC**	sodium dimethyldithiocarbamate.
**SpTS**	sodium p-toluenesulfonate.
**SD**	standard deviation.
**SE**	standard error.
**SSB**	DNA single-strand breaks.
**Sudan**	sudan I: 1-phenylazo-2-naphthol.
**ZDEDC**	zinc diethyldithiocarbamate*.
**ZDMDC**	zinc dimethyldithiocarbamate.
**ZnCl_2_**	zinc chloride.
**ZPS**	zinc phenolsulfonate*.
**ZpTS**	zinc p-toluenesulfonate hydrate*.
*****	identified as data-poor chemicals.
