# Supplementary material for: Effects of Copper or Zinc Organometallics on Cytotoxicity, DNA Damage and Epigenetic Changes in the HC-04 Human Liver Cell Line
Source: Int J Mol Sci. 2023 Oct 25;24(21):15580. doi: 10.3390/ijms242115580 (PMC10650525; doi:10.3390/ijms242115580)
Supplement: Supplementary file 1 [file ijms-24-15580-s001.zip › ijms-2635323-supplementary.pdf]

Desaulniers et al Supplement

Effects of copper or zinc organometallics on cytotoxicity, DNA damage and epigenetic changes in the HC-04 human liver cell line.

Daniel Desaulniers, Gu Zhou, Andrew Stalker, Cathy Cummings-Lorbetskie.

Contents

DNA DAMAGE ASSAYS 2

γH2AX assay 2

DNA unwinding assay 3

Figure S1. Activity of cytochrome p450-1A1 (CYP1A1) and CYP3A4 in HepG2 and HC-04 cells exposed to aflatoxin-B1. 5

Figure S2. Method used to derive NOELs and LOELs using the data generated by aflatoxin-B1.. 6

Figure S3. Validation of the multiplex bead array approach to measure changes in histone modifications in HC-04 cells by testing the effects of 5 h exposure to the histone deacetylase inhibitor trichostatin-A (TSA........ 7

Figure S4. Validation of the multiplex bead array approach to measure changes in the abundance of H3K27Me3 and H3K9Me3 in HC-04 cells by testing the effects of 48h exposure to solvent control (SC) or to the histone methyltransferase inhibitor GSK-126. The grey bars indicate the net MFI of total H3. 8

Figure S5. Flow cytometry analysis of global genome changes in DNA methylation induced by 24h incubation with 0 µM (A,B) 5 µM (C, D) and 50 µM (E, F) DNA methyltransferase inhibitor 5-aza-2'-deoxycytidine (positive control). 10

Figure S6. Concentration-response effects of 24h exposure to the DNA methyltransferase inhibitor 5-aza-2'-deoxycytidine (5aCdR) on global genome DNA methylation. 11

Figure S7. Concentration-response effects of 24h exposure to copper dimethyldithiocarbamate (CDMDC) on global genome DNA methylation. 12

Figure S8. Concentration-response effects of 72h exposure to copper-dimethytldithiocarbamate CDMDC) on global genome DNA methylation. 13

Figure S9. No observable effect levels (NOELs: black circle, and open square) and lowest observable effect levels (LOELs: open circle and triangle) inducing an increase in DNA abundance. 14

Figure S10. Global genome DNA methylation obtained by multiplying by 100 the ratio of the 5-methyl-cytosine fluorescence signal divided by the DNA abundance fluorescence signal. 15

DNA DAMAGE ASSAYS

γH2AX assay

The measurement of phosphorylated H2AX (pH2AX) provides an indication of the functionality of enzymatic systems at detecting and signaling the presence of DNA damage. DSBs activate the ataxia-telangiectasia mutated (ATM) gene and the DNA-dependent protein kinase catalytic subunit (DNA–PKcs), which phosphorylate multiple DNA repair proteins, including histone H2AX. Phosphorylated H2AX provides a binding site for MDC1 (Mediator of DNA Damage Checkpoint 1), which promotes spreading of pH2AX (then called γH2AX) for hundreds of kilobases on either side of the break. DSBs also promote complex patterns of chromatin ubiquitination, including ubiquitination of H2A/H2AX by the RNF8/RNF168 (Ring finger protein 8/168) ubiquitin ligases, which, in turn, creates binding sites for repair proteins such as 53BP1 (p53-binding protein 1) and BRCA1 (Breast cancer gene 1) [reviewed in [1]. The pH2AX can then be removed (via H2A.X–H2B dimers) by FACT (Facilitates chromatin transcription complex) and replaced with canonical H2A–H2B dimers [2;3].

The adhering cells were fixed and permeabilized in the wells using proprietary reagents supplied with the commercial ELISA assay kit "Human/Mouse/Rat Phospho-Histone H2AX (S139) Immunoassay" (R&D systems, Minneapolis, MN). The cells were simultaneously incubated with two primary antibodies raised in different species: a phospho-specific antibody and a normalization antibody that recognizes the total H2AX proteins regardless of phosphorylation status. Two secondary antibodies specific to the species of origin of the primary antibodies and labeled with either horseradish-peroxidase or alkaline phosphatase, and two spectrally distinct fluorogenic substrates, constitute the detection system.

HC-04 cells were harvested at 70-80% confluency and seeded at a density of 1.5×10^4 cells/100 µl/well in 96-well culture plates coated with collagen (Sigma-Aldrich Canada Co. Oakville, Ontario), and incubated overnight at 37°C and 5% CO_2. Test chemical solutions were prepared at double the test concentrations in medium and were added to each well at a volume of 100 µl. Three wells with cells received only the secondary antibody as negative control, and two wells without cells were used as background control. Chemicals were tested in triplicate and incubated for 24 h. The cells were fixed and permeabilized in the wells. The cells were simultaneously incubated with two primary antibodies overnight at 4°C. The culture wells were washed and incubated for two hours at room temperature (RT) with the two secondary antibodies (including the negative control wells). The culture wells were washed and incubated at RT for 60 min with the first fluorescent substrate. The second fluorescent substrate was added and incubated at RT for an additional 30 min. The plates were examined for fluorescence with wavelength for excitation (ex) at 540 nm and emission (em) at 600 nm for the pH2AX proteins, and ex = 360 nm, em = 450 nm for total H2AX proteins (Synergy 2 Multi-Mode Reader, BioTek, Winooski, VT).

After reading the fluorescence values for the γH2AX assay, the plates were washed four times with 200 µl PBS, each time on a platform shaker for 5 min. Absence of residual

fluorescence from the γH2AX assay was confirmed. The DNA-specific fluorescent dye Hoechst 33258 (Sigma) [4], was added to each well (5 µg/ml in PBS; 50 µl/well) and incubated at RT for 5 min. Fluorescence was read at 346/461 nm excitation/emission (Synergy 2 Multi-Mode Reader, BioTek, Winooski, VT USA). Background reading (wells with no dye) was subtracted from all wells, and treated to control well reading ratios were used to derive toxicity index values (expressed as percentages).

DNA unwinding assay

HC-04 cells were seeded (10,000/well) in a sterile 96-well opaque plate and cultured overnight before treating with increasing concentrations of positive control substances [hydrogen peroxide (30 min), or 4-nitroquinoline-N1-oxide (30 min or 24 h), or AFB1 (30 min or 24 h)] or test chemicals (24 h). After treatment, the wells were washed with PBS, exposed to 50 µL of PicoGreen solution (1% v/v PicoGreen, 0.05% Triton-X100 in Ca/Mg-free PBS), and incubated at room temperature covered with tinfoil for 30 min. Prior to initiating DNA unwinding, four fluorescence pre-readings (485/538 nm excitation/emission, Spectramax M2 Fluorometer, Molecular Devices, Sunny Vale, CA) at 30 s intervals were taken to ensure complete dye penetration and thus stable readings. These pre-unwinding readings differ across wells and provide an index of the amount of DNA in each well, thus the fourth pre-reading value was used to normalize the subsequent unwinding values. Then, DNA unwinding was initiated by adding freshly prepared unwinding solution (20 mM EDTA, 74 mM NaOH; 80 to 90 µL depending on the experiment) to titrate the final medium to pH 12.3. Note that the required volumes of solution required to reach pH 12.3 was verified using other flasks immediately prior to the experiments. For example, if the volume of unwinding buffer required to bring 5 mL of PBS (with 1% DMSO and 0.05% triton to match the PicoGreen solution composition) to pH 12.3 was 8.3 mL, then 83 µl of unwinding buffer was added to the wells of the assay. Fluorescence in the culture wells were read immediately after the addition of the unwinding solution and then at 1 min intervals.

The pre-unwinding fluorescence reading (pre-read) represents total amount of double stranded DNA at the beginning. This amount differs across wells depending on (1) the accuracy of cell seeding, (2) proliferation differences occurring over the culture period prior and during treatment, (3) detachment of cells due to toxicity over the treatment period, and (4) elimination of dead and floating cells through washing procedures prior to the unwinding assay. Thus, the pre-read fluorescence was used as an index of treatment toxicity and reflects abundance of remaining attached cells from which the Strand Scission Factor (SSF) was calculated.

The Strand Scission Factor (SSF) was calculated after 20 min of denaturation, as performed by others [5], using the following formula for each culture well:

Normalized fluorescence of a sample (NFs):

$$\frac{\text{Fluorescence of a sample at T = 20 min} - \text{background}}{\text{Pre-read fluorescence of the sample at T = 0 min.} - \text{background.}}$$

Normalized fluorescence of solvent control (NFc):

Fluorescence of a solvent control at T = 20 min – background

Pre-read fluorescence of solvent control at T = 0 min. –background.

$$SSF\ (T = 20\ min) = -1(\log_{10}(NFs/NFc))$$

The ratio NFs/NFc is smaller than 1, and thus the Log_{10} (NFs/NFc) generates negative values that are multiplied by -1 to obtain positive numbers where increasing values indicate increasing amount of damaged DNA relative to control samples. Note that values for each well are reported relative to the average NFc of the control wells (minimum in triplicate). If cells within a well have less damaged DNA than in the average of the controls, then the SSF will become a negative value.

Figure S1. Activity of cytochrome p450-1A1 (CYP1A1) and CYP3A4 in HepG2 and HC-04 cells exposed to aflatoxin-B1.

Cells (10,000/wells, 96 well plate) were seeded in HC-04 medium (described in the main paper), or HepG2 medium (Eagles's minimum essential medium, 10% fetal bovine serum, penicillin-streptomycin (100 U and 100 µg per mL, 1% GlutaMaxTM, reagents from Life Technologies)). 24 h later the medium was replaced with media containing increasing concentrations of aflatoxin-B1 (10 µM DMSO as vehicle). The assays were conducted 72h later following the manufacturer instructions of the P450-Glo™ CYP1A1 assay, P450-Glo™ CYP3A4 assay with Luciferin-IPA, and ATP CellTiterGlo® Luminescent Cell Viability Assay, all from Promega Corp. (Madison, WI, USA). The Y-axis reveals major differences in basal and aflatoxin-B1-induced enzyme activities. *: statistically significant increase relative to control (0 µM), Dunnett's method $p < 0.05$. Means + SD, n=3.

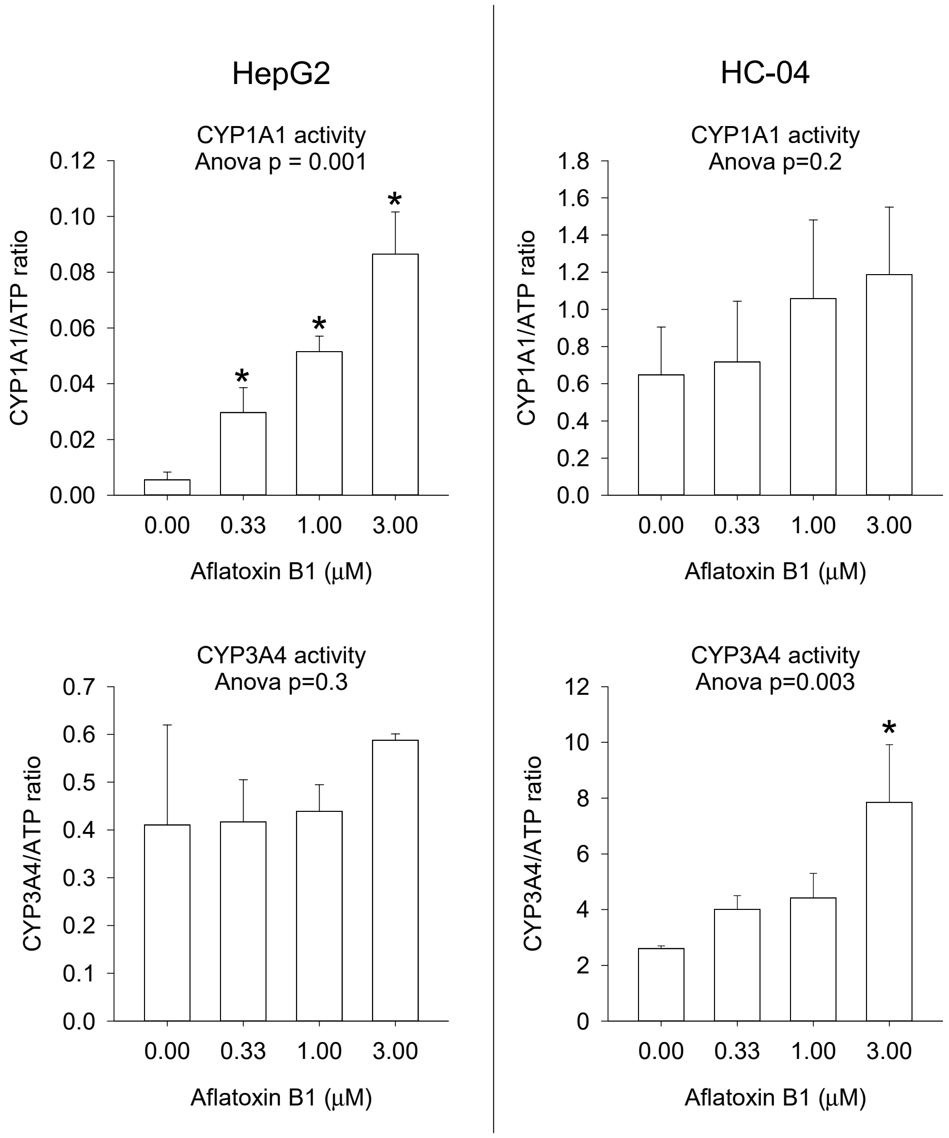

Figure S2. Method used to derive NOELs and LOELs using the data generated by aflatoxin-B1.

These results are from cells exposed to AFB1 for 24h with assays performed 24h after cessation of exposure. A 24h delay prior to performing the assay appeared to increase the sensitivity of the assay. This effect was also observed while testing 4-nitroquinoline-oxide. The NOELs and LOELs were transferred to Fig. 1, 2 and 3, in the main document. For example, in the graph pH2AX/DNA, the NOEL was 0.63 µM, and the LOEL is 1.25 µM. Occasionally, negative values are generated by the formula of the SSF (page 4) when no DNA damages are induced. Within each experiment the treatments were represented by 4 to 6 wells (2 wells in some rare cases). The data sets for all experiments were combined and analysed by two-way ANOVA considering the effects of experiments, chemical concentrations, and the interaction experiments X chemical concentrations (details in the data analyses section). Instead of presenting a typical experiment, the graphs show the least square means + SE of all the data form all experiments. Means with different letters are significantly different, Tukey's HSD $p<0.05$.

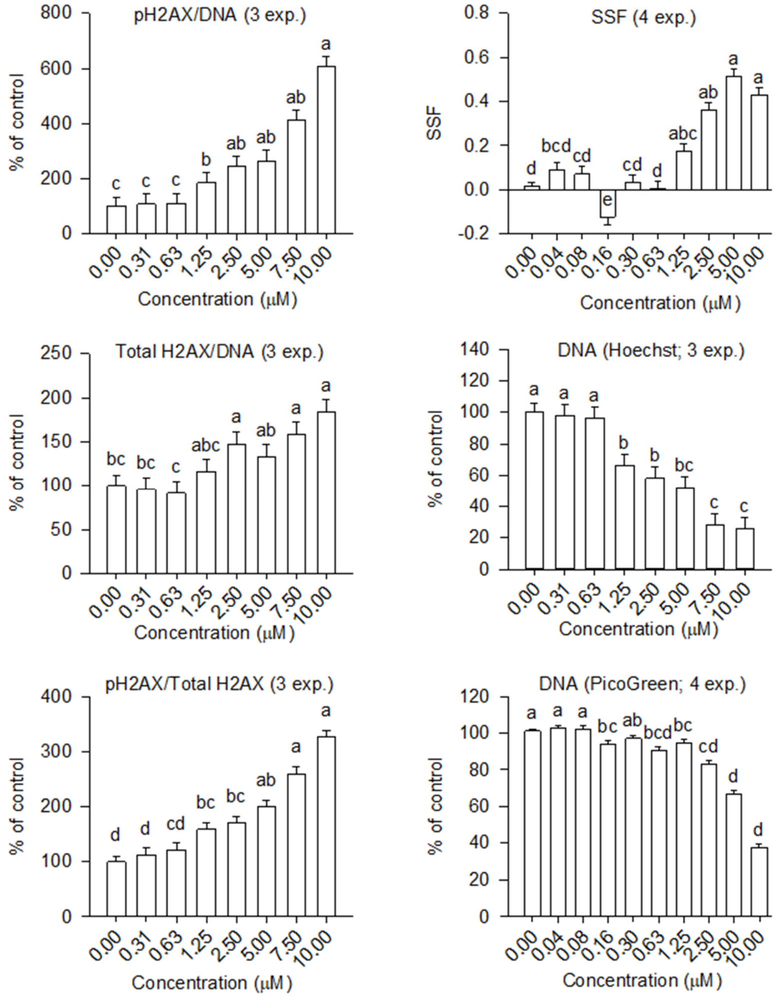

Figure S3. Validation of the multiplex bead array approach to measure changes in histone modifications in HC-04 cells by testing the effects of 5 h exposure to the histone deacetylase inhibitor trichostatin-A (TSA). The net median fluorescence intensity (MFI) of each H3 modification was measured relative to the net MFI of total histone. Mean ± SD, n=2. *: significant difference from the solvent control (SC), Dunnett's method p<0.05.

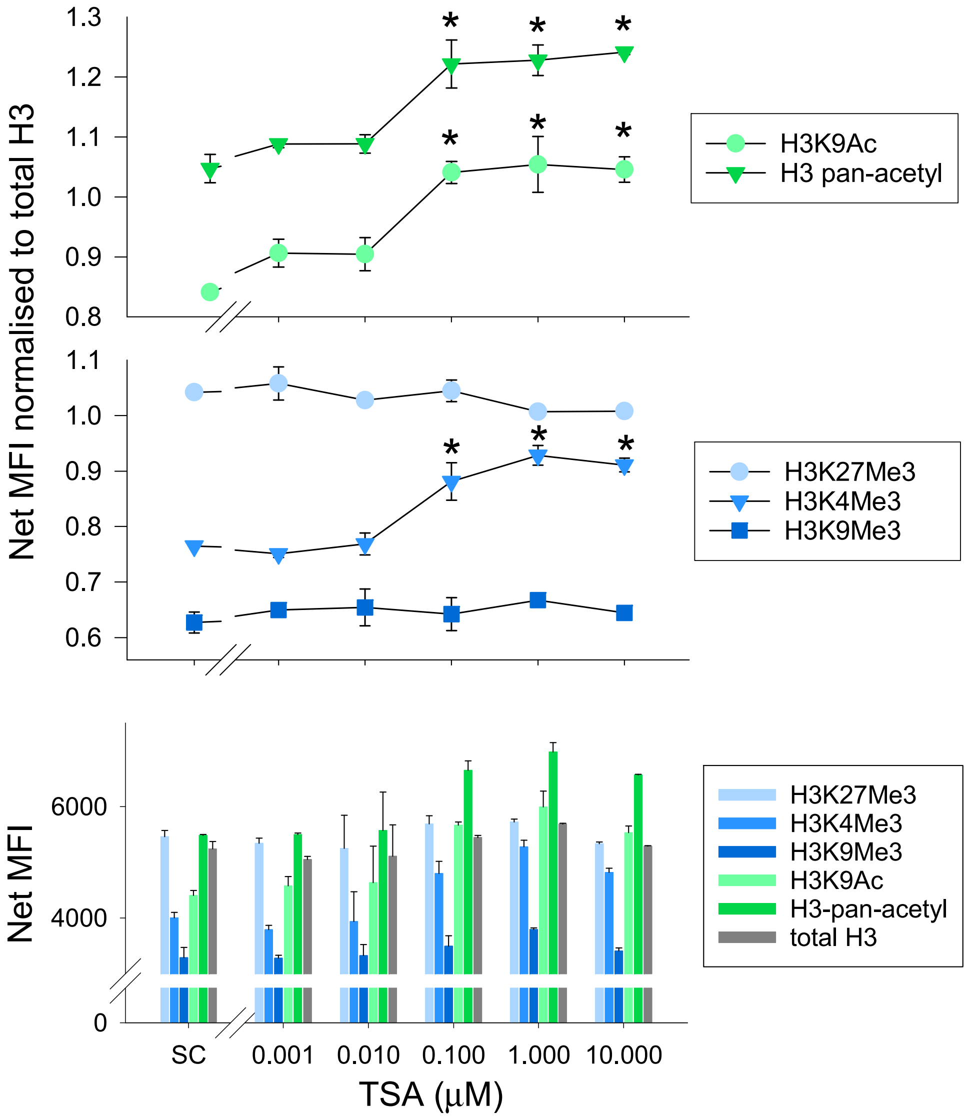

As expected, TSA increased H3 acetylation but interestingly also increased the abundance of H3K4me3 (Figure S3). These observations were confirmed in a second experiment where the abundance of H3K9Ac, H3 pan-acetyl, and H3K4me3 relative to H3 were increased ($p<0.05$) by 5 h exposure to 2 µM TSA (1.27 ± 0.02, 1.53 ± 0.04, 0.745 ± 0.02, respectively) compared to control samples (0.67 ± 0.17, 0.85 ± 0.15, 0.44 ± 0.19, respectively) (mean ± SD, n=3).

Figure S4. Validation of the multiplex bead array approach to measure changes in the abundance of H3K27Me3 and H3K9Me3 in HC-04 cells by testing the effects of 48h exposure to solvent control (SC) or to the histone methyltransferase inhibitor GSK-126. The grey bars indicate the net MFI of total H3. Mean ± SD, n=3. *: ANOVA on rank followed by Dunnett's method, $p<0.05$.

GSK126 (GlaxoSmithKline 126) is a highly selective inhibitor of EZH2 (Enhancer of zeste homolog 2; a histone-lysine N-methyltransferase enzyme). EZH2 is the catalytic subunit of the polycomb repressive complex 2 (PRC2) methylating histone H3 on lysine 27 (H3K27). Despite that it is acting as competitive substrate for the universal methyl donor, S-adenosylmethionine (SAM), the GSK126 treatments specifically decrease abundance of methylated H3K27, an observation previously reported in lymphoma cells [6].

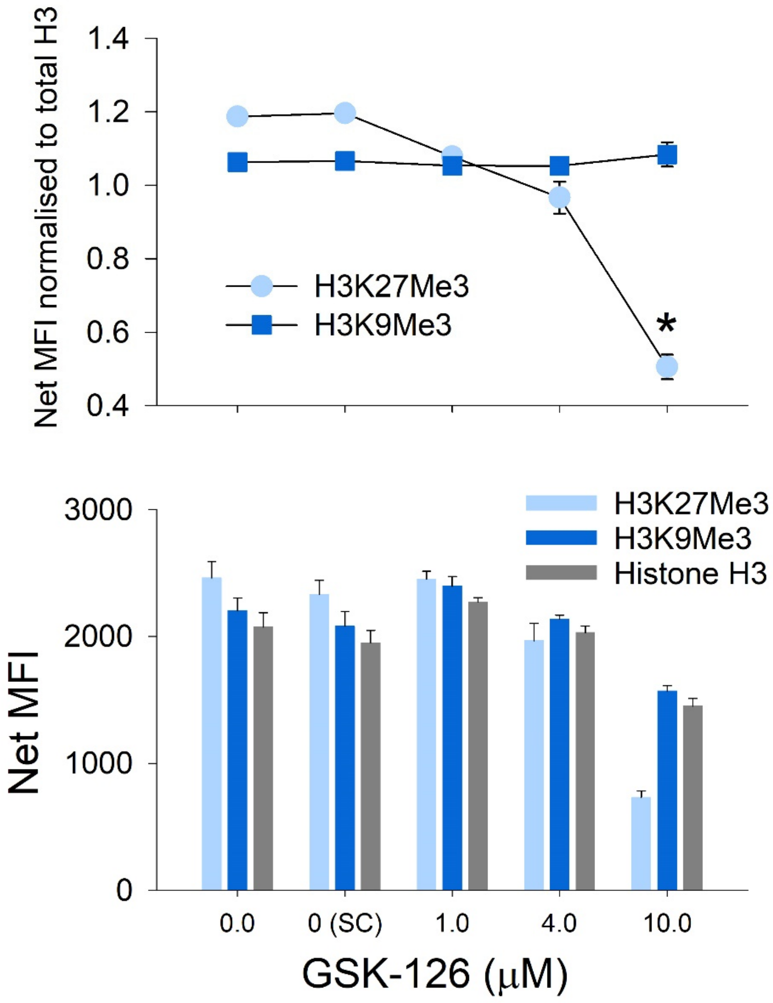
Net MFI normalised to total H3
1.4
1.2
1.0
0.8
0.6
0.4
H3K27Me3
H3K9Me3
*
3000
2000
1000
0
Net MFI
H3K27Me3
H3K9Me3
Histone H3
0.0
0 (SC)
1.0
4.0
10.0
GSK-126 (μM)

Figure S5. Flow cytometry analysis of global genome changes in DNA methylation induced by 24h incubation with 0 µM (A,B) 5 µM (C, D) and 50 µM (E, F) DNA methyltransferase inhibitor 5-aza-2'-deoxycytidine (positive control). Propidium iodide staining for DNA content (Y-axis: PI-A) permitted to separate the analyses of cell population at the G1 and G2 phases of the cell cycle (e.g.: 61.7%, 23.7% and 3.14% of the cells were in G1, G2, or supra-G2 at 0 µM, respectively). The Supra-G2 cells were not further analysed. Graphs B, D, F show the frequency distribution of G1 cells based on the DNA methylation signal intensity (FITC-A signal on the X-axis) derived from a fluorescent labelled antibody directed at methylated cytosines. The gates to quantify the number of cells in the left (hypomethylation) and right tail (hypermethylation) of the distribution were fixed based on the average of the medians and SD of control samples. For example, at 0 µM and 5 µM 1.4% and 56.1% of the cells were hypomethylated, respectively. Two populations of G1 cells are clearly visible at 50 µM (E, F).

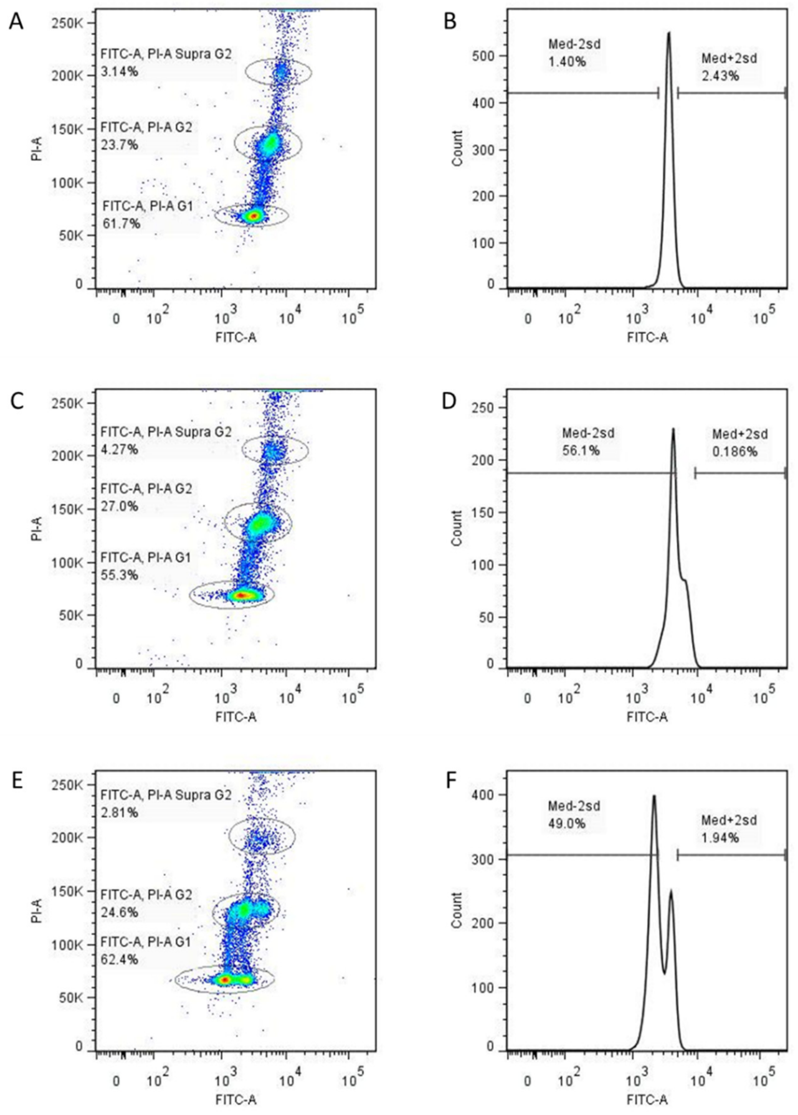

Figure S6. Concentration-response effects of 24h exposure to the DNA methyltransferase inhibitor 5-aza-2'-deoxycytidine (5aCdR) on global genome DNA methylation. The related percent changes in DNA methylation are presented in Figure S10.

A) Ploidy explains the signal intensity differences between the G1 and G2 cells. A first significant decreases in the median fluorescence intensity of G1 (diploid) and G2 (tetraploid) cells occurred at 0.5 µM 5aCdR. B) The percentage of hypomethylated cells in the left tail of the distributions of G1 and G2 cells increased at 5 µM (G2 analysed on log transformed data). Both A and B indicate hypomethylation with increasing concentrations of 5aCdR in absence of overt toxicity which occurred at 50 µM; 94%, 86%, and 68% of the cells were counted in the 0.5, 5.0, and 50 µM treatment groups relative to the vehicle (final concentration with less than 0.0125% acetic acid; stock solution at 100 mM in 25% acetic acid). C) The percentages of cells measured in the right distribution tail of the distribution were small and significantly reduced at 0.5 µM but only in the G2 cells. The experiment was repeated twice with 2 to 3 samples per treatment group in each experiment. The data were analysed by two-way ANOVA considering effects of concentration, experiment, and their interactions. *: significant difference from vehicle group (acetic acid), Dunnett's method $p < 0.05$. Least square mean + SE of the data from both experiments.

	Conc.	Exp.	Conc. X Exp.
Median G1	<0.001	0.002	0.005
Median G2	<0.001	<0.001	0.002
Left tail G1	<0.001	0.84	<0.001
Left tail G2	<0.001	0.06	0.41
Right tail G1	0.13	0.94	0.05
Right tail G2	0.03	0.7	0.05

A) Methylation median of G1 and G2 cells

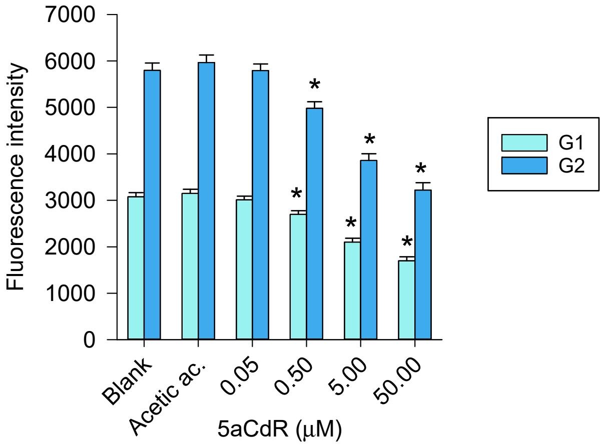

B) Percentages of cells in the left distribution tail.

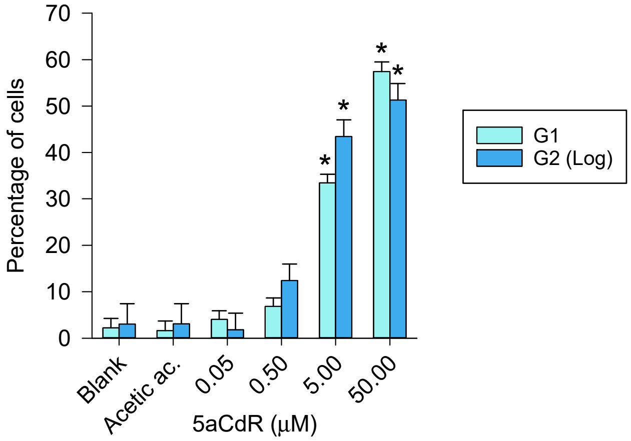

C) Percentages of cells in the right distribution tail.

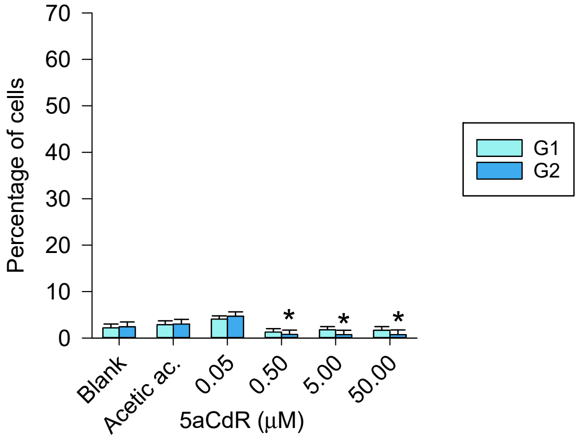

Figure S7. Concentration-response effects of 24h exposure to copper dimethyldithiocarbamate (CDMDC) on global genome DNA methylation. One experiment, n=2, each are shown in the graphs. *: first concentration inducing a significant difference relative to DMSO, Dunnett's method p < 0.05.

A) Median methylation signal intensity of G1 and G2 cell populations.

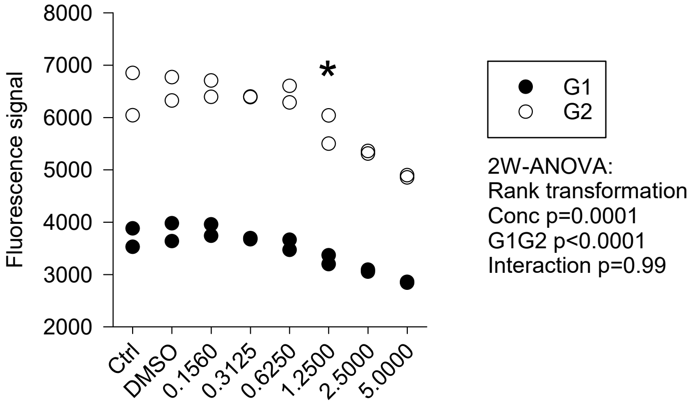

B) Percent of G1 and G2 cells in left distribution tails.

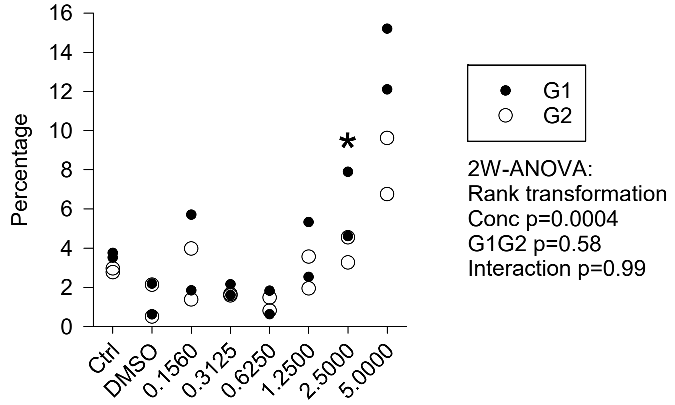

C) Percent of G1 and G2 cells in right distribution tails.

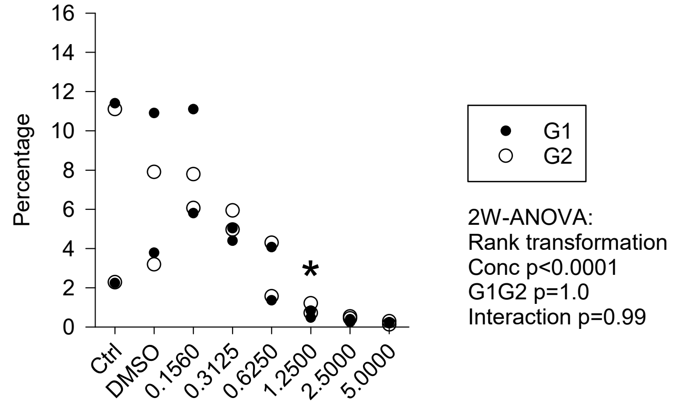

Figure S8. Concentration-response effects of 72h exposure to copper-dimethytldithiocarbamate CDMDC) on global genome DNA methylation.

Despite that 0.3125 µM CDMDC reduced the number of cells to 33% of the vehicle DMSO group, there were no effect of treatments on the (A) median signal intensity, and percentage of cells in the (B) left, and (C) right distribution tails of the G1 and G2 cells. The experiment was repeated twice with 2 to 3 samples per treatment group in each experiment. The data were analysed by two-way ANOVA considering effects of concentration, experiment, and their interactions. Although the G2 methylation median (A) showed an effect of concentration with p=0.04, the multiple comparison procedure (Dunnett's method) did not detect any statistically significant effect.

	Conc.	Exp.	Conc. X Exp.
Median G1	0.25	0.009	0.54
Median G2	0.04	0.21	0.05
Left tail G1	0.96	0.003	0.04
Left tail G2	0.99	0.005	0.02
Right tail G1	0.96	0.04	0.11
Right tail G2	0.96	0.008	0.002

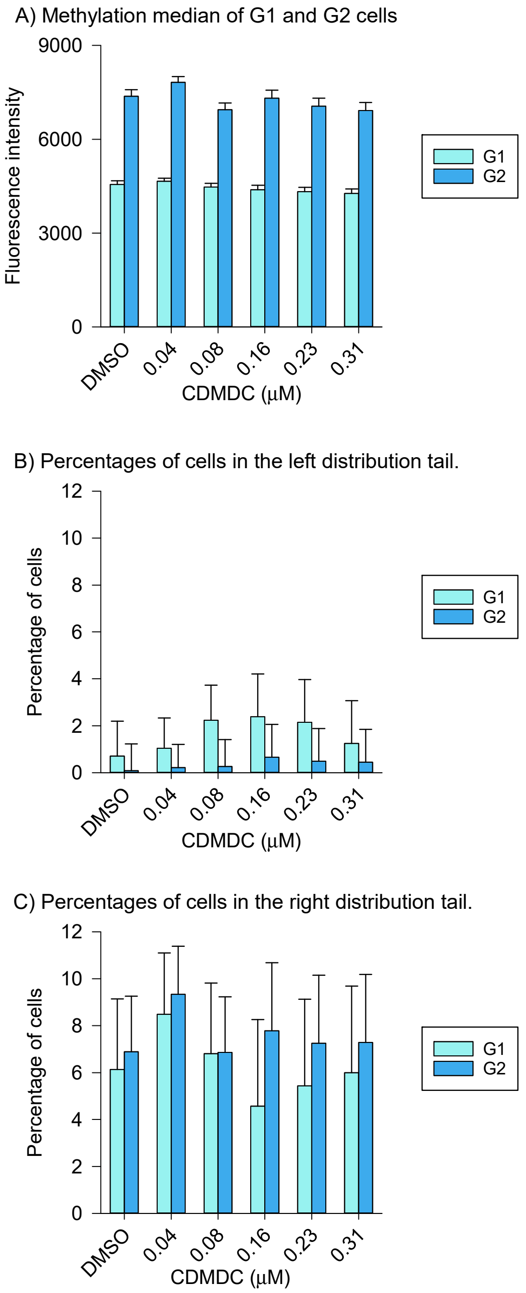

Figure S9. No observable effect levels (NOELs: black circle, and open square) and lowest observable effect levels (LOELs: open circle and triangle) inducing an increase in DNA abundance. This graph is presented because some chemicals induced increases in DNA abundance prior to decreases DNA abundance as indication of toxicity. Data obtained from Hoechst or PicoGreen DNA staining were from the γH2AX or SSF assays, respectively. Some data were from only the Hoechst (e.g. CuDg), or only the PicoGreen assays (e.g. BDMP3CZ). The largest concentration tested is provided within parenthesis beside the chemical name. No NOEL symbols are provided when the LOEL was the smallest concentration tested. Chemicals in red are the "data poor" chemicals.

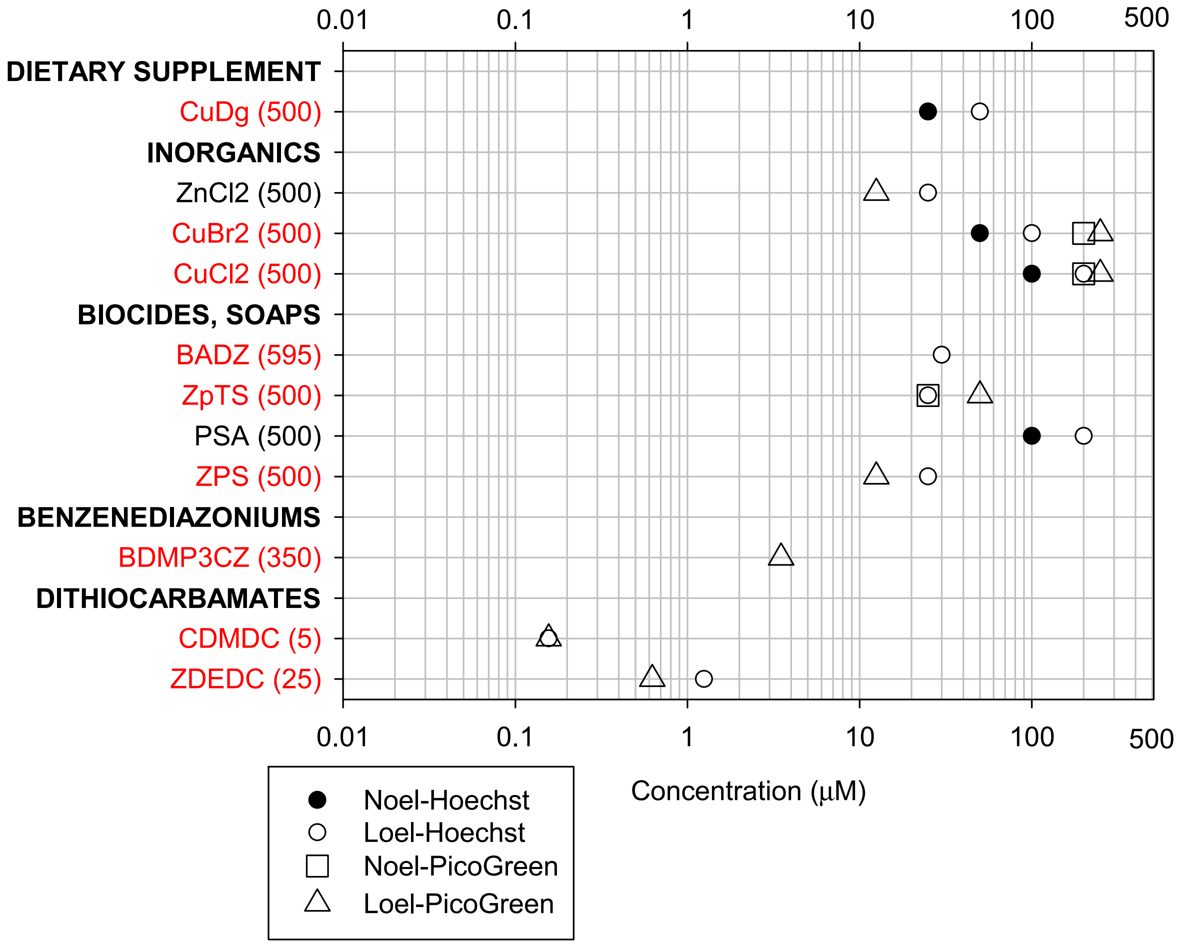

Figure S10. Global genome DNA methylation obtained by multiplying by 100 the ratio of the 5-methylcytosine fluorescence signal divided by the DNA abundance fluorescence signal (propidium iodide signal).

The data from the experiments of figure 8, 9, and S6, are presented relative to the average of the G1 and G2 data across experiments to generate means of 100% in the control groups (0, and DMSO). Blank refers to untreated cells. A) $CuCl_2$ increased methylation at 25 µM, but 100 to 500 µM decreased methylation relative to control (0). B) ZDMDC at concentrations ≥0.63 µM increased methylation relative to the vehicle group (DMSO). C), 5aCdR increased methylation at 0.05 µM, followed by decreases at 0.5, 5, and 50 µM. Means + SE. Means with different letters are significantly different, Tukey's HSD p<0.05.

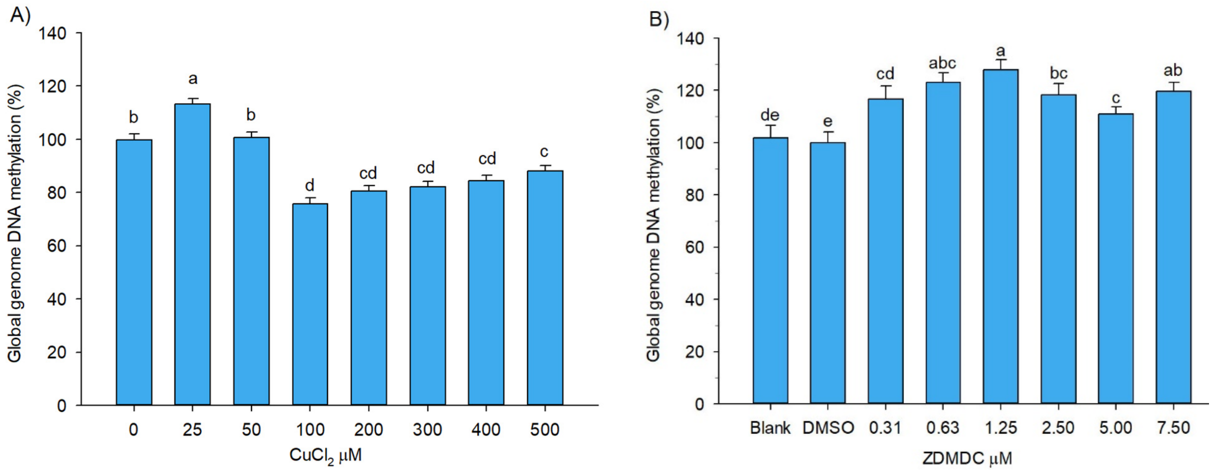

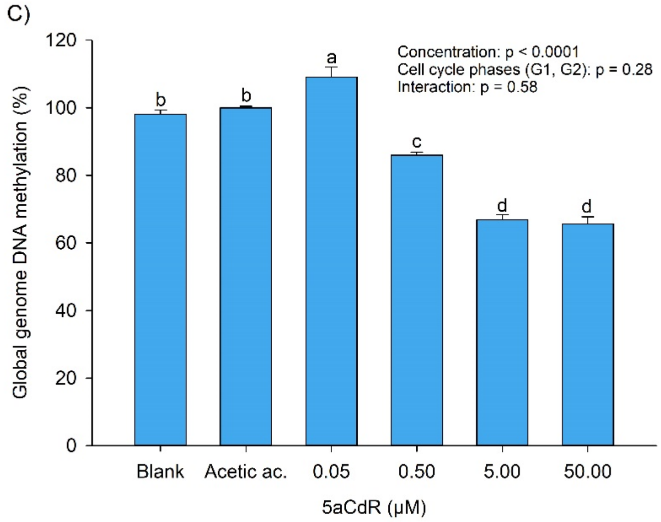

Source	A) CuCl2 Prob > F	B) ZDMDC Prob > F
Conc:	<.0001	<.0001
Phase:	0.79	0.61
Exp:	0.0002	<.0001
Conc*Phase:	0.98	0.96
Exp*Phase:	0.34	0.99
Exp*Conc:	<.0001	<.0001
Exp*Conc*Phase:	0.59	1.00

Reference List

1. Gursoy-Yuzugullu, O.; Ayrapetov, M.K.; Price, B.D.: Histone chaperone Anp32e removes H2A.Z from DNA double-strand breaks and promotes nucleosome reorganization and DNA repair. *Proc Natl Acad Sci U S A* **2015**;*112*:7507-7512.

2. Heo, K.; Kim, H.; Choi, S.H.; Choi, J.; Kim, K.; Gu, J.; Lieber, M.R.; Yang, A.S.; An, W.: FACT-mediated exchange of histone variant H2AX regulated by phosphorylation of H2AX and ADP-ribosylation of Spt16. *Mol Cell* **2008**;*30*:86-97.

3. Dahlin, J.L.; Chen, X.; Walters, M.A.; Zhang, Z.: Histone-modifying enzymes, histone modifications and histone chaperones in nucleosome assembly: Lessons learned from Rtt109 histone acetyltransferases. *Crit Rev Biochem Mol Biol* **2015**;*50*:31-53.

4. Begg, A.C.; Mooren, E.: Rapid fluorescence-based assay for radiosensitivity and chemosensitivity testing in mammalian cells in vitro. *Cancer Res* **1989**;*49*:565-569.

5. Batel, R.; Jaksic, Z.; Bihari, N.; Hamer, B.; Fafandel, M.; Chauvin, C.; Schroder, H.C.; Muller, W.E.; Zahn, R.K.: A microplate assay for DNA damage determination (fast micromethod). *Anal Biochem* **1999**;*270*:195-200.

6. McCabe, M.T.; Ott, H.M.; Ganji, G.; Korenchuk, S.; Thompson, C.; Van Aller, G.S.; Liu, Y.; Graves, A.P.; Della, P.A., III; Diaz, E.; LaFrance, L.V.; Mellinger, M.; Duquenne, C.; Tian, X.; Kruger, R.G.; McHugh, C.F.; Brandt, M.; Miller, W.H.; Dhanak, D.; Verma, S.K.; Tummino, P.J.; Creasy, C.L.: EZH2 inhibition as a therapeutic strategy for lymphoma with EZH2-activating mutations. *Nature* **2012**;*492*:108-112.
